# A Highly Conserved Bacterial D-Serine Uptake System Links Host Metabolism and Virulence

**DOI:** 10.1371/journal.ppat.1005359

**Published:** 2016-01-04

**Authors:** James P. R. Connolly, Mads Gabrielsen, Robert J. Goldstone, Rhys Grinter, Dai Wang, Richard J. Cogdell, Daniel Walker, David G. E. Smith, Andrew J. Roe

**Affiliations:** 1 Institute of Infection, Immunity and Inflammation, College of Medical, Veterinary and Life Sciences, University of Glasgow, Glasgow, United Kingdom; 2 Cancer Research UK Beatson Institute, Glasgow, United Kingdom; 3 School of Life Sciences, Heriot-Watt University, Edinburgh, United Kingdom; 4 Department of Microbiology, Monash University, Melbourne, Victoria, Australia; 5 State Key Laboratory of Molecular Vaccinology and Molecular Diagnostics, School of Public Health, Xiamen University, Xiamen, Fujian, People's Republic of China; 6 Institute of Molecular, Cell and Systems Biology, College of Medical, Veterinary and Life Sciences, University of Glasgow, Glasgow, United Kingdom; University of Utah, UNITED STATES

## Abstract

The ability of any organism to sense and respond to challenges presented in the environment is critically important for promoting or restricting colonization of specific sites. Recent work has demonstrated that the host metabolite D-serine has the ability to markedly influence the outcome of infection by repressing the type III secretion system of enterohaemorrhagic *Escherichia coli* (EHEC) in a concentration-dependent manner. However, exactly how EHEC monitors environmental D-serine is not understood. In this work, we have identified two highly conserved members of the *E*. *coli* core genome, encoding an inner membrane transporter and a transcriptional regulator, which collectively help to “sense” levels of D-serine by regulating its uptake from the environment and in turn influencing global gene expression. Both proteins are required for full expression of the type III secretion system and diversely regulated prophage-encoded effector proteins demonstrating an important infection-relevant adaptation of the core genome. We propose that this system acts as a key safety net, sampling the environment for this metabolite, thereby promoting colonization of EHEC to favorable sites within the host.

## Author Summary

The host intestinal tract is a challenging environment for any foreign organisms not usually found within the microbiota. Intruding pathogens must survive physical barriers and readily compete with the local micro flora for limited nutrients within their preferred niche. In order to do this, intestinal pathogens have adapted many methods of sensing the environment for numerous chemical signals that may be encountered, and thus translate these signals to the relevant transcriptional networks that promote colonization. However, intestinal pathogens will also encounter a vast variety of signals both prior to entry into the intestinal tract and post fecal shedding. Therefore sensing specific signals from these environments is key to limiting the colonization of unfavorable environments. In this work we have elaborated on previous findings that the host metabolite D-serine, found in abundance at extraintestinal sites, can repress colonization of enterohaemorrhagic *Escherichia coli* (EHEC), by demonstrating a mechanism used to sample D-serine from the environment. Responding appropriately to host-relevant signals is critically important for niche recognition and also reveals the adaptive power of bacterial pathogens. Knowledge of such signals reveals the detailed mechanisms about niche adaptation, how colonization is regulated and could potentially be used for the design of novel intervention strategies to limit intestinal pathogenesis.

## Introduction

*Escherichia coli* is an extremely diverse Gram-negative bacterial species, commonly establishing itself as a commensal member of the microbiota early after birth in healthy humans and animals [1]. However, owing to a large degree of genome plasticity, numerous pathogenic forms of *E*. *coli* dubbed ‘pathotypes’ have emerged and can be classified broadly according to the site of the body in which they cause infection [2–4]. Pathotypes largely associated with enteric illness include enterohaemorrhagic *E*. *coli* (EHEC), enteropathogenic *E*. *coli* (EPEC), enteroaggregative *E*. *coli* (EAEC), enterotoxigenic *E*. *coli* (ETEC), enteroinvasive *E*. *coli* (EIEC) and diffusely adherent *E*. *coli* (DAEC), which are responsible for varying degrees of diarrheal disease by unique mechanisms. Extraintestinal pathogenic *E*. *coli* (ExPEC) colonise and compete in the gastrointestinal tract but have the capacity to disseminate to distal sites. Mostly notably these include urinary tract pathogenic *E*. *coli* (UPEC) and the lesser-explored meningitis associated *E*. *coli* (MNEC) [4–6].

Classically, tropism to a particular site has largely been attributed to the specificity of bacterial adhesins to host receptors. Arguably the best example is Type 1 fimbriae, responsible for binding to D-mannose residues on mucosal cells that are central to UPEC adhesion and virulence [7]. However, receptor complement varies and adhesins are often highly immunogenic and therefore their production is a tightly regulated process [8]. Recent work has highlighted the importance of host metabolites in modulating the expression of bacterial colonization factors at sites of infection [9]. EHEC (most notably the O157:H7 serotype) is an aggressive food-borne isolate that primarily colonizes the terminal rectum of ruminants asymptomatically and the large intestine of humans, resulting in haemorrhagic colitis from very low infectious doses and in extreme cases can cause fatal haemolytic uremic syndrome [10,11]. Fucose, a major component of mucin glycoproteins and abundant energy source in the large intestine, is sensed by EHEC using a two-component sensory system, FusKR, which in turn affects both virulence gene expression and colonization [12]. Similarly, ethanolamine a major component of both bacterial and mammalian cell membranes is not only important for nitrogen metabolism but is also used as a molecule in cell-to-cell signaling to activate virulence gene expression in the gastrointestinal tract [13]. The presence of particular metabolites has also been reported to act as repressive signals that limit colonization in unfavorable environments. Biotin, an essential co-factor found in abundance in the small intestine, has been recently shown to inhibit EHEC colonization at this site by modulating global gene expression thus promoting passage towards the large intestine where EHEC favorably colonizes and where biotin concentrations are much reduced [14]. These studies demonstrate an emerging understanding of how specific pathotypes sense and respond to the minutiae of conditions present at various sites encountered within the host whilst highlighting the mechanistically diverse nature of this process.

Colonization of EHEC is mediated by a type III secretion system (T3SS) encoded on a large pathogenicity island (PAI) known as the locus of enterocyte effacement (LEE). The LEE-encoded T3SS allows intimate attachment to host cells mediating the attaching and effacing (A/E) phenotype, a hallmark of LEE-associated pathogens [15]. The EHEC LEE encodes 41 open reading frames (ORFs) mostly across five polycistronic operons, named *LEE1* through *LEE5* [16]. As well as encoding the necessary machinery and primary effectors of this T3SS, the LEE also carries two master regulatory systems that control LEE expression at the core level–the LEE encoded regulator (Ler) and the global regulator of Ler activation (GrlA) [17–20]. Key research investigating physiologically relevant signals that EHEC encounter during infection has revealed the LEE as a hub for environmental sensing and thus control of colonization within the correct niche [9].

We recently reported that the host metabolite D-serine could selectively inhibit expression of the LEE-encoded T3SS in EHEC [21]. D-serine is a metabolite found in abundance at various extraintestinal sites in the human body, including the urinary tract and the brain, and modulates virulence gene expression of ExPEC such as the UPEC strain CFT073 whilst also being used as a carbon source [22–24]. This is achieved by the conversion of D-serine to pyruvate and ammonia via the D-serine tolerance locus, *dsdCXA* [25,26]. This locus is truncated however in many intestinal strains of *E*. *coli* resulting in an inability to metabolize D-serine and rendering them susceptible to the toxic effects of this amino acid [27]. Intriguingly, we found that the ability to metabolize D-serine did not alleviate inhibition of the LEE, thus separating the virulence and metabolic phenotypes and offering an explanation as to why EHEC isolates have not acquired this pathogenicity island [21]. Our results suggested that acquisition of the LEE is a key factor in restricting the individual to the intestinal tract where inhibitory molecules such as D-serine are in sub-inhibitory abundance and is an example of niche adaptation for this pathotype.

D-serine represses virulence through modulation of the LEE transcriptional network [21]. However, the precise mechanism by which D-serine was specifically sensed by EHEC remained obscure. In this study we have identified a D-serine sensory locus. This system includes a D-serine inner membrane transporter, YhaO, and a LysR-type transcriptional regulator, YhaJ, that are both required for full virulence in EHEC. We reveal that YhaO is a functional D-serine transporter in both EHEC and UPEC but is regulated uniquely in each background, and that YhaJ is required for its expression under LEE-inducing conditions (growth in MEM-HEPES at 37°C). Furthermore, we demonstrate that YhaJ directly regulates Ler expression by enhancing activation of the major LEE promoter. These genes are highly conserved across the *E*. *coli* phylogeny but this work demonstrates the adaptive capacity of the *E*. *coli* core genome towards new and important functions. We propose that YhaO has been recruited by EHEC upon acquisition of the LEE to maintain an active response system capable of transporting and sensing environmental D-serine, thus ensuring appropriate colonization factor expression and in turn, niche specificity of this pathotype.

## Results

### Identification of a highly conserved putative D-serine metabolic locus

**Fig 1:**
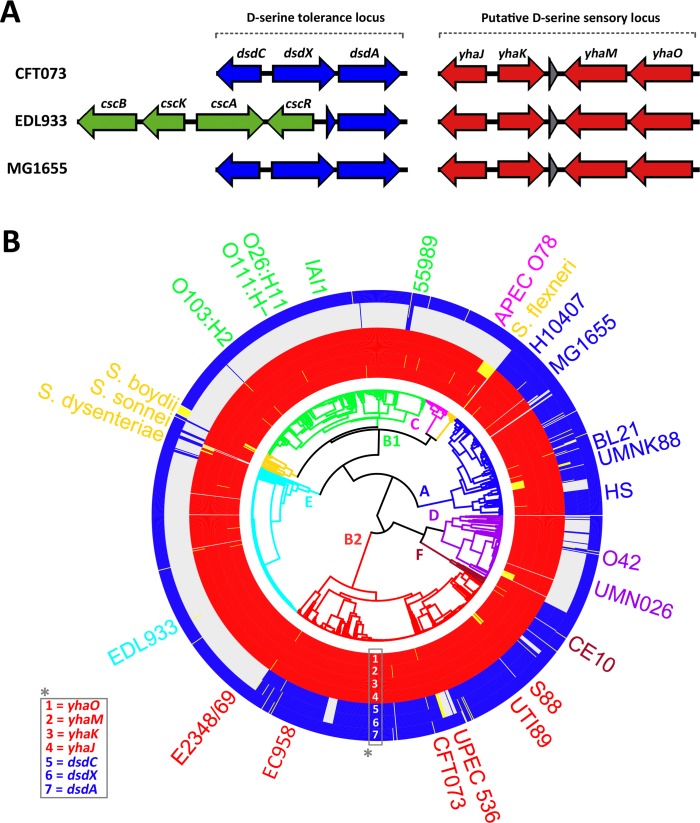
Genomic and phylogenomic context of the *yhaOMKJ* locus. (A) Genomic context of the D-serine tolerance locus (blue) in three distinct *E*. *coli* isolates–CFT073 (UPEC), EDL933 (EHEC) and MG1655 (K-12). The system encodes DsdC (a LysR type transcriptional regulator), DsdX (a D-serine outer membrane transporter) and DsdA (a D-serine deaminase). In EDL933 the D-serine tolerance locus is truncated and replaced with the sucrose utilization locus (*cscRAKB* highlighted in green). (B) Genomic context of the second putative D-serine sensory locus (red) in CFT073, EDL933 and MG1655. The system encodes YhaJ (a putative LysR type transcriptional regulator), YhaK (a redox-sensitive bicupin), YhaM (a putative deaminase) and YhaO (a putative inner membrane D-serine transporter). (B) The *yhaOMKJ* locus is highly conserved across the *E*. *coli* phylogeny. Circularized phylogenomic tree of 1591 *E*. *coli* and *Shigella* isolates overlaid with gene carriage for the *dsdCXA* locus and the *yhaOMKJ* locus. The *yhaOMKJ* genes are indicated by red blocks and the *dsdCXA* locus by blue blocks. Ordering of the genes is numbered and corresponds to the gene in the legend labeled *. Presence of a gene is determined by > 80% identity over > 80% of the coding sequence. Pseudogenes are indicated as yellow blocks. *E*. *coli* phylogroups are subdivided by color with the branch point labeled on the tree. Phylogroup A = Blue; Phylogroup B1 = Green; Phylogroup B2 = Red; Phylogroup C = Magenta; Phylogroup D = Purple; Phylogroup E = Cyan; Phylogroup F = Brown; *Shigella* = Gold. The position of prototypical strains is indicated on the outside of the figure.

Our previous work had identified D-serine as a key host metabolite affecting EHEC colonization and niche specificity [21]. However, how D-serine was sensed by EHEC remained an unanswered question. Exposure to D-serine results in activation of the SOS stress response and repression of the LEE-encoded T3SS. Based on this aforementioned data, we postulated a sensing mechanism would be co-expressed with the LEE to facilitate detection of environmental D-serine during colonization of host tissue. Investigation of published microarray data during growth in MEM-HEPES (that induces maximal LEE expression *in vitro*) revealed genes that were co-regulated with the LEE [28]. This subset of genes was screened using Pfam to identify any exhibiting possible functions associated with serine metabolism or transport. The most highly upregulated gene was *yhaO* (Z4463), encoding an uncharacterized putative serine inner membrane transporter, displaying a 43 fold increase in expression (P ≤ 0.0005). Adjacent to *yhaO* are *yhaM* (Z4462), encoding a putative serine dehydratase and *yhaJ* (Z4459) a LysR-type transcriptional regulator (LTTR) (Fig 1A). This cluster of genes was of interest as its genetic organization and putative functions were similar to that of the D-serine tolerance locus from UPEC, *dsdCXA* (Fig 1A), encoding a D-serine transporter, DsdX, and a D-serine dehydratase, DsdA, both of which are regulated by a LTTR, DsdC [23,26]. A further gene, *yhaK* (Z4460), encoding a redox-sensitive bicupin that is also positively regulated in human urine, a D-serine-rich environment, is oriented divergently from *yhaJ* (Fig 1A) [29,30]. Reverse transcriptase-PCR analysis of these genes confirmed their transcription under LEE-inducing conditions and also identified co-transcription of *yhaO* and *yhaM* in a similar manner to *dsdX* and *dsdA* from CFT073 (S1 Fig). D-serine is considered toxic to certain *E*. *coli* isolates that lack *dsdCXA*, including EHEC [27,31]. This made the discovery of *yhaOMKJ* highly intriguing and begged numerous questions about its functionality and role in EHEC.

Our previous investigation into the effects of D-serine on virulence gene expression in EHEC revealed that carriage of *dsdCXA* was widespread primarily across extraintestinal isolates but also commonly found in intestinal isolates that do not carry the LEE PAI [21]. We used this comparative genomics approach here to investigate if *yhaOMKJ* is restricted to any particular lineage(s) of *E*. *coli*. We compared the carriage of *yhaOMKJ* and *dsdCXA* using the presence or absence approach in 1581 unique genome sequences of *E*. *coli* and the closely related *Shigella* sp. In contrast to *dsdCXA*, which is carried in 38% of genomes (the vast majority being LEE-negative), *yhaOMKJ* is highly conserved across the entire *E*. *coli* phylogeny with all four genes being retained in over 94% genomes (Fig 1B). A low frequency of pseudogene appearances occur in the genomes analyzed for *yhaO*, *yhaM* and *yhaJ* (3.29%, 0.44% and 1.83% respectively). Attrition in *yhaJ* shows a preference within *Shigella* isolates (S1 Table). Of the 1581 genomes analysed, 86% of *yhaJ* pseudogenes were classified as *Shigella* isolates. Indeed, over half of the *Shigella* genomes analysed carried a *yhaJ* pseudogene, an intriguing finding given that the differentiation between these two species is largely based on their pathogenicity and antigenicity rather than genetic distinction [32]. The ancestral nature of this locus and its high degree of conservation suggest an important role in *E*. *coli*. More specifically, in EHEC, the inability to grow on D-serine as a sole carbon source implies that YhaO and YhaJ cannot be simply involved in the metabolism of this amino acid but rather that they have been adapted for another as yet undefined function.

### Identification of YhaO and YhaJ as virulence determinants in EHEC

**Fig 2:**
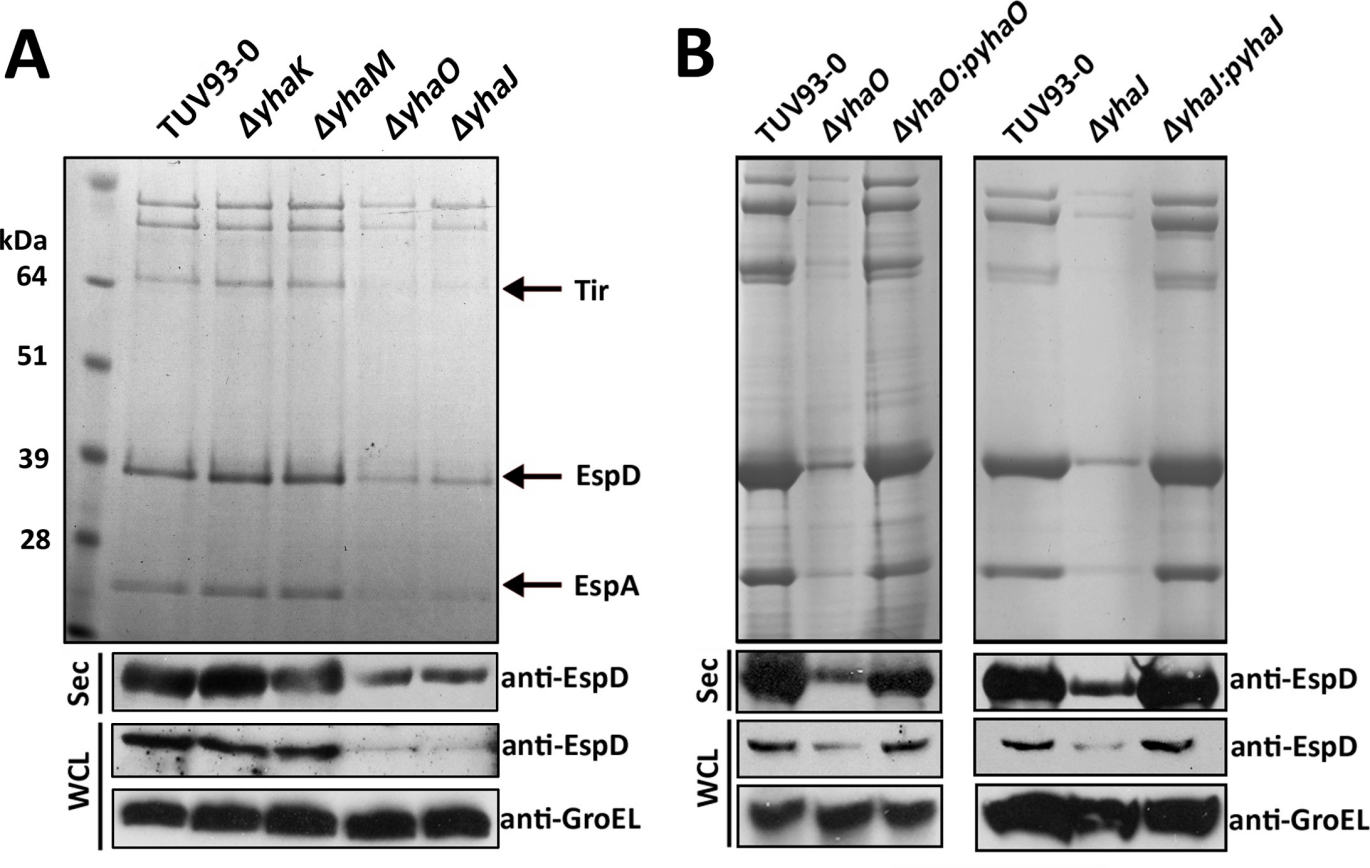
Identification of YhaO and YhaJ as potential virulence determinants. (A) Screening of the *yhaOMKJ* locus for a role in virulence. SDS-PAGE profile of secreted proteins from TUV93-0, *yhaO*, *yhaM*, *yhaK* and *yhaJ* cultured in MEM-HEPES. Arrows indicate the location of the major LEE-encoded secreted effectors Tir, EspD and EspA as identified by mass-spectrometry. Samples were normalized according to cellular OD^600^ to normalize loading into each well. Immunoblot analysis of EspD levels from secreted (Sec) and whole cell lysate (WCL) fractions confirmed the SDS-PAGE results. Anti-GroEL was used to verify equal concentrations of WCL, which corresponded to OD^600^ normalized culture samples, loaded into each well (B) SDS-PAGE analysis highlighting complementation of the *ΔyhaO* and *ΔyhaJ* phenotypes by plasmids p*yhaO* and p*yhaJ*. SDS PAGE and immunoblot analysis of secreted protein profiles and EspD cytoplasmic expression confirmed the results. Protein secretion experiments were performed on multiple occasions.

Previous work has shown the importance of the *dsdCXA* locus for virulence gene expression in UPEC [22,24,33]. We therefore tested whether the *yhaOMKJ* genes affected expression of key virulence factors in EHEC. Non-polar deletion mutants for each of the four genes were generated and tested for effects on secretion of LEE-encoded effector proteins. SDS-PAGE analysis showed that *yhaO* and *yhaJ* were required for full activity of the LEE-encoded-T3SS, with a marked reduction in the secretion of associated proteins including Tir, EspD and EspA. Immunoblot analysis of secreted EspD confirmed the phenotypes of the *ΔyhaO* and *ΔyhaJ* mutants showing an ~3.2 and ~2.6 fold reduction in secreted EspD compared to the wild type. Immunoblot analysis of EspD from whole cell lysates also confirmed that not just secretion but also production of EspD was reduced in the *ΔyhaO* and *ΔyhaJ* mutant backgrounds. Deletion of *yhaK* or *yhaM* did not affect T3SS-associated protein production or secretion (Fig 2A). To verify the phenotypes observed were the consequence of the specific deletions, complementation was achieved by transformation with a plasmid-borne copy of the appropriate gene, fully restoring the production and secretion of effector proteins (Fig 2B). The mutants showed no change in growth rate in minimal media or any significant alterations in motility when inoculated onto soft agar plates (S2 Fig).

### RNA-seq analysis of *ΔyhaO* and *ΔyhaJ* under LEE-inducing conditions

**Fig 3:**
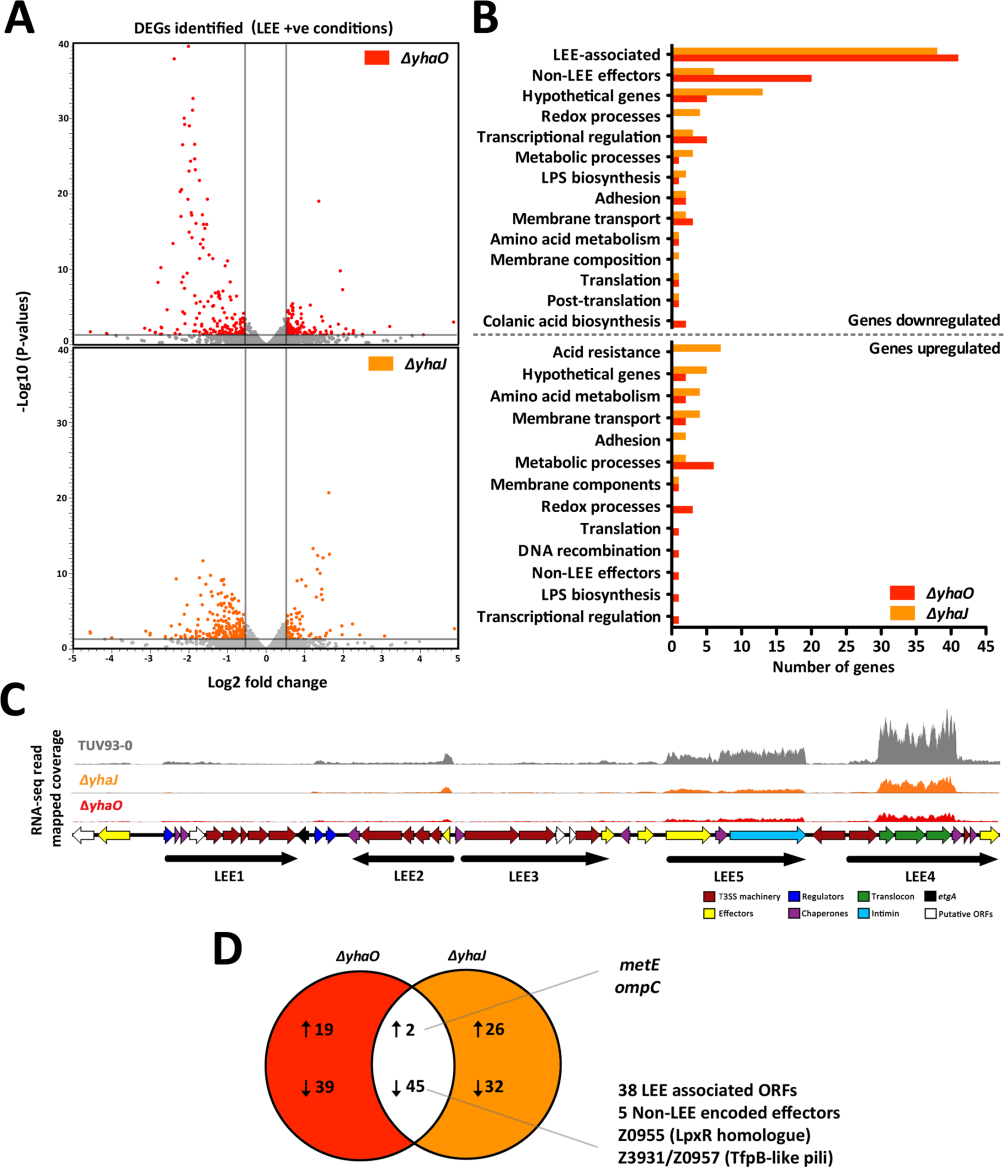
RNA-seq analysis of *ΔyhaO* and *ΔyhaJ* under LEE-inducing conditions. (A) Volcano plots illustrating the identification of differentially expressed genes (DEGs) in the *ΔyhaO* (red) and *ΔyhaJ* (orange) mutants when grown in MEM-HEPES to induce expression of the LEE. Grey bars indicate the cutoffs for DEG identification (corrected P-values on the Y-axis and fold change on the X-axis). (B) Functional grouping of DEGs identified according to broad GO terms. Read and orange bars correspond to wild type TUV93-0 versus *ΔyhaO* and *ΔyhaJ* respectively. The numbers of DEGs for each group are indicated below and the graph is separated according to downregulated genes and upregulated genes. (C) RNA-seq read coverage mapping across the LEE pathogenicity island illustrating downregulation of the LEE in the *ΔyhaO* and *ΔyhaJ* mutants under inducing conditions. Coverage is color coded as grey (TUV93-0), red (*ΔyhaO*) and orange (*ΔyhaJ*). Maximum height of the read peaks has been scaled according to the wild type TUV93-0. Genetic organization of the LEE has been indicated below so as to correspond to coverage peaks. Individual ORFs are color coded according to the legend and the operon structure of the LEE is indicated in black. (D) Venn diagram indicating the overlap between the *ΔyhaO* and *ΔyhaJ* regulons. Upward or downward arrows indicate upregulated and downregulated DEGs respectively. The DEGs found in both datasets are annotated on the right of the figure.

To examine the role of YhaO and YhaJ under LEE-inducing conditions we examined the global effects caused by deletion of *yhaO* and *yhaJ* using comparative RNA-sequencing analysis (RNA-seq). This revealed 105 differentially expressed genes (DEGs; defined as fold-change greater than 1.5 and an FDR corrected P-value of less than 0.05) between the wild type TUV93-0 and *ΔyhaO* mutant (Fig 3A). Broad functional grouping according to the literature and gene ontology (GO) annotation revealed that more than half of the DEGs identified were involved directly in virulence (Fig 3B). The entire LEE PAI was significantly downregulated, which is comprised of 41 ORFs and includes the master regulators *ler* and *grlA*. Twenty non-LEE encoded effectors (NLEs), encoded across diverse elements of the EHEC genome, were also decreased as well as three putative adhesins (Fig 3B; S3 Fig). For the 21 genes that were upregulated their functions were highly varied with roles in cellular metabolism, cell envelope biogenesis and bacteriophage-associated genes. Analysis of the *ΔyhaJ* mutant revealed 103 DEGs from the wild type, of which 77 were down-regulated and 29 up-regulated displaying diverse functions (Fig 3B). Of the down-regulated genes, again over half were associated with the LEE (38 ORFs) with a further 5 genes encoding NLE proteins. This pattern of expression was mirrored in the *ΔyhaO* mutant data set (Fig 3C; S4 Fig). The transcriptomic profiles of *ΔyhaO* and *ΔyhaJ* showed very extensive overlap in virulence-related ORFs but also showed unique effects on global gene expression in either background (Fig 3D). In both mutants, 45 genes were down-regulated including 38 LEE associated genes, 5 genes encoding NLE proteins and an LpxR homologue (Z0955) suggested to play a role in pathogenesis by modulating lipid-A and, hence, cytokine responses [34]. The RNA-seq data were validated by qRT-PCR analysis for five genes (*espD*, *tir*, *ler*, *nleA* and *nleG*) identified as being differentially expressed by RNA-seq (S5 Fig). These data identified YhaO and YhaJ as being part of very specific regulons primarily involved in LEE-associated pathogenesis however the differential regulation of a number of non-virulence associated genes in both backgrounds suggests that YhaO and YhaJ likely have other significant and independent roles depending on the conditions tested. RNA-Seq data are summarized in S2 Table.

### YhaO and YhaJ are required for maximal A/E lesion formation on host cells

**Fig 4:**
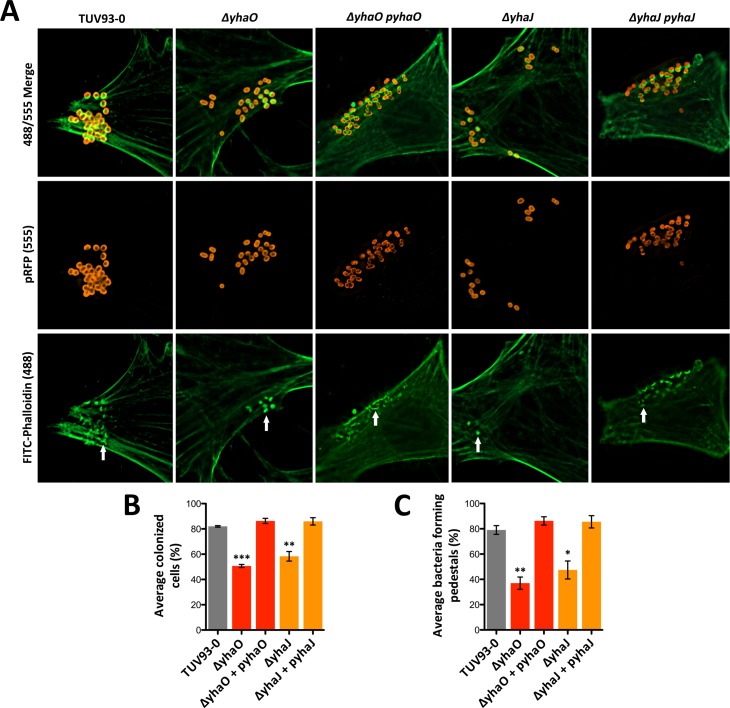
YhaO and YhaJ are required for attaching and effacing lesion formation on host cells. (A) Wide-field fluorescence microscopy images of HeLa cells incubated with TUV93-0, *ΔyhaO*, *ΔyhaO* + p*yhaO*, *ΔyhaJ* and *ΔyhaJ* + p*yhaJ* in MEM-HEPES (LEE-inducing conditions). Host cells were stained with FITC-Phalloidin to fluorescently label actin green (488) and bacterial cells were either transformed with a plasmid constitutively expressing RFP (*ΔyhaO* and *ΔyhaJ*) or stained with Alexafluor 555 (p*yhaO* and p*yhaJ*) to label them red. Merged channels clearly show the areas of localized actin condensation beneath colonized bacterial cells, which corresponds to A/E lesion and pedestal formation as indicated by a white arrow. (B) Quantification of the average percentage of colonized host cells in the *ΔyhaO* and *ΔyhaJ* mutants and corresponding complementation backgrounds relative to TUV93-0. (C) Quantification of the average percentage of attached bacteria forming A/E lesions on bound host cells. Data was calculated from three biological replicates with at least twenty-five random fields of view taken per replicate. ***, ** and * denote P ≤ 0.001, P ≤ 0.01 and P ≤ 0.05 respectively.

Given that deletion of *yhaO* or *yhaJ* selectively down-regulated the T3SS, we tested the ability of EHEC to bind to host cells and intimately attach via A/E lesions. EHEC use the LEE-encoded T3SS to translocate effector proteins into the host cell resulting in A/E pedestal formation and distinctive areas of condensed host-cell actin (Fig 4A). Deletion of either *yhaO* or *yhaJ* resulted in significantly (P ≤ 0.001 and P ≤ 0.01 respectively) fewer infected host cells relative to the TUV93-0 control in which over 80% of host cells imaged were colonized. Complementation of either mutant (p*yhaO* and p*yhaJ*) restored host cell attachment to that of wild type levels (Fig 4B). Moreover, for both mutants, the proportion of remaining attached bacteria formed significantly fewer pedestals (P ≤ 0.01 and P ≤ 0.05 respectively) than the wild type, which were identified by areas of actin condensation, a phenotype that is consistent with reduced effector protein production. This was in contrast with the WT EHEC strain, for which approximately 80% of attached bacteria formed pedestals. Similarly to the proportion of colonized host cells, complementation of either mutant resulted in pedestal formation comparable to the wild type (Fig 4C).

### YhaO transports D-serine in both EHEC and UPEC

**Fig 5:**
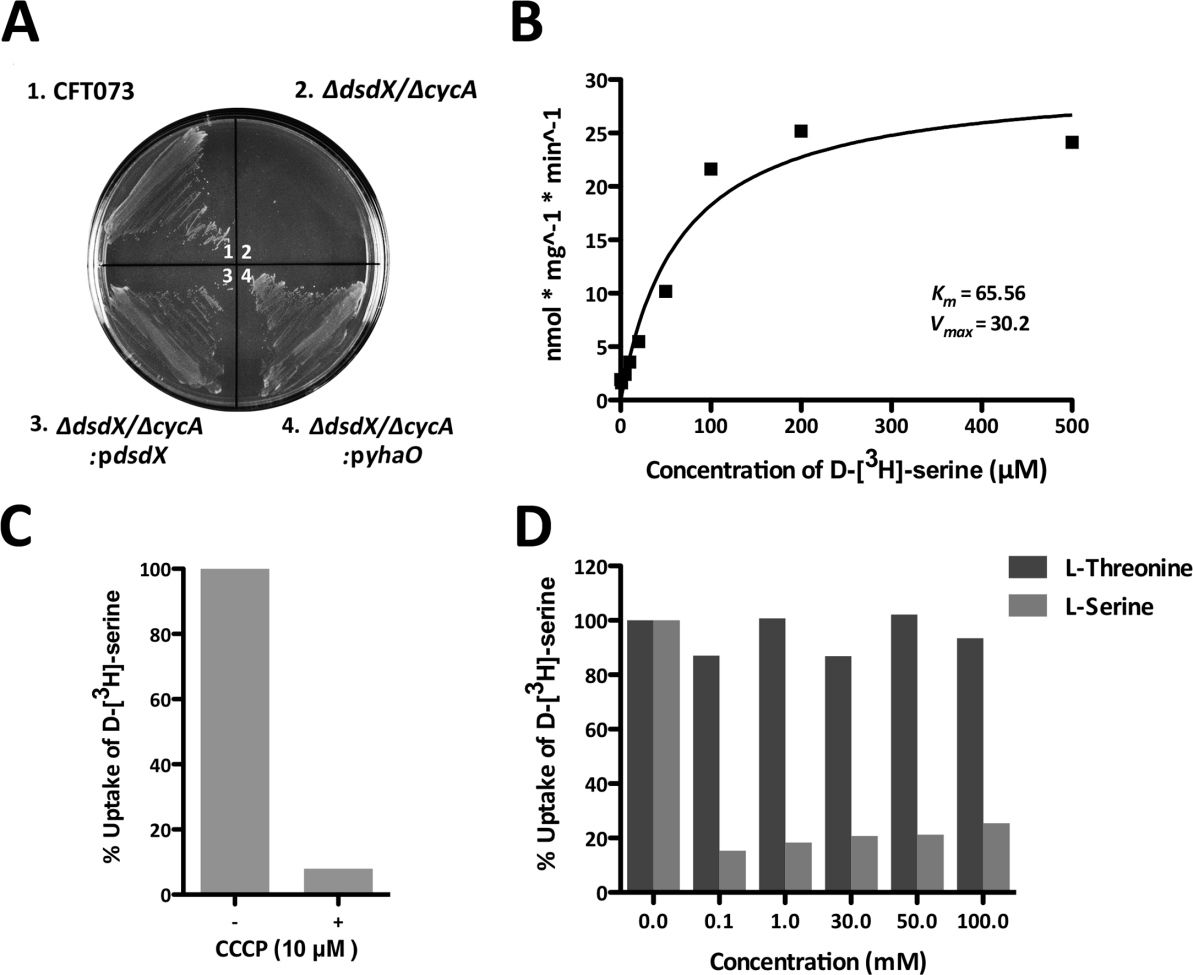
Biochemical analyses of YhaO functionality in a CFT073 *ΔdsdXΔcycA* background. (A) Assessment of the ability of YhaO to transport D-serine. Comparison of the UPEC wild type CFT073 (1) and the D-serine transporter mutant *ΔdsdXΔcycA* (2) for the ability to grow on MOPS minimal agar plates containing D-serine as a sole carbon source. Complementation of *ΔdsdXΔcycA* with either p*dsdX* from CFT073 (3) or p*yhaO* from TUV93-0 (4) restored the ability to grow on D-serine as a carbon source. (B) Concentration dependent uptake of D-[^3^H]-serine. A concentration range of 0 to 200 μM D-[^3^H]-serine was tested and uptake was represented as nmol*mg^-1^*min^-1^. (C) Addition of 10 μM CCCP to the reaction (+) at 100% relative uptake of D-[^3^H]-serine was used to determine the effects of membrane potential on D-[^3^H]-serine uptake by YhaO. (D) Competitive uptake of L-serine and L-threonine by YhaO. A concentration range (0 to 100 mM) L-serine or L-threonine were added to the reaction at 100% relative uptake of D-[^3^H]-serine to determine the specificity of YhaO for D-serine. Bars representing L-threonine and L-serine are indicated in dark and light grey respectively.

Having established that both YhaO and YhaJ play roles in modulating virulence gene expression we aimed to understand better their functions in order to offer mechanistic insight into this phenomenon. YhaO is predicted to be an inner membrane serine/threonine transporter. To examine if YhaO was capable of transporting serine, we used a UPEC *ΔdsdXΔcycA* mutant that has previously been reported to be unable to transport D-serine and consequently fails to grow on MOPS minimal agar plates containing this amino acid as a sole carbon source (Fig 5A) [23]. Growth could be restored by complementation with a plasmid over-expressing DsdX, the characterized D-serine transporter from UPEC CFT073 or by expression of YhaO from EDL933, strongly supporting the notion that YhaO is capable of transporting D-serine. To further investigate this, radiolabeled D-[^3^H]-serine was then used to examine D-serine transport in the same genetic background. Uptake of D-[^3^H]-serine was measured over a range of concentrations from 0.01 μM to 200 μM and determined following 5 min incubation. Increasing the external D-[^3^H]-serine concentration from 0.01 μM to 100 μM resulted in a linear increase within the bacterial cells. Concentrations of D-[^3^H]-serine above 100 μM did not give any further accumulation (Fig 5B). To confirm that the uptake of D-[^3^H]-serine is related to active transport rather than non-specific binding to the cell surface, uptake was measured in the absence and presence of 10 μM carbonyl cyanide m-chlorophenylhydrazone (CCCP), used to uncouple the H^+^-gradient of the cell. The uptake was reduced by greater than 90% when CCCP was present, showing that the transport of D-serine is reliant on the H^+^-gradient (Fig 5C). To determine the specificity of the transporter, competition experiments were performed in which increasing concentrations of unlabeled amino acids were pre-incubated in the uptake assay before D-[^3^H]-serine was added. L-threonine, a similar hydroxyl amino acid to D-serine affected uptake minimally (less than 10% reduction compared to the control samples), even at concentrations of up to 100 μM, suggesting poor affinity for L-threonine. In contrast, addition of L-serine at 0.01 μM resulted in an 85% reduction of D-[^3^H]-serine uptake (Fig 5D). Together, these results demonstrate that YhaO is capable of transporting D-serine but that it is not capable of discriminating between the two isomeric forms of this amino acid. These data are intriguing considering that the co-transcribed YhaM cannot restore growth to a UPEC *ΔdsdA* mutant or facilitate EHEC survival on D-serine as a carbon source, calling into question the functionality of YhaM as a serine deaminase (S6 Fig).

**Fig 6:**
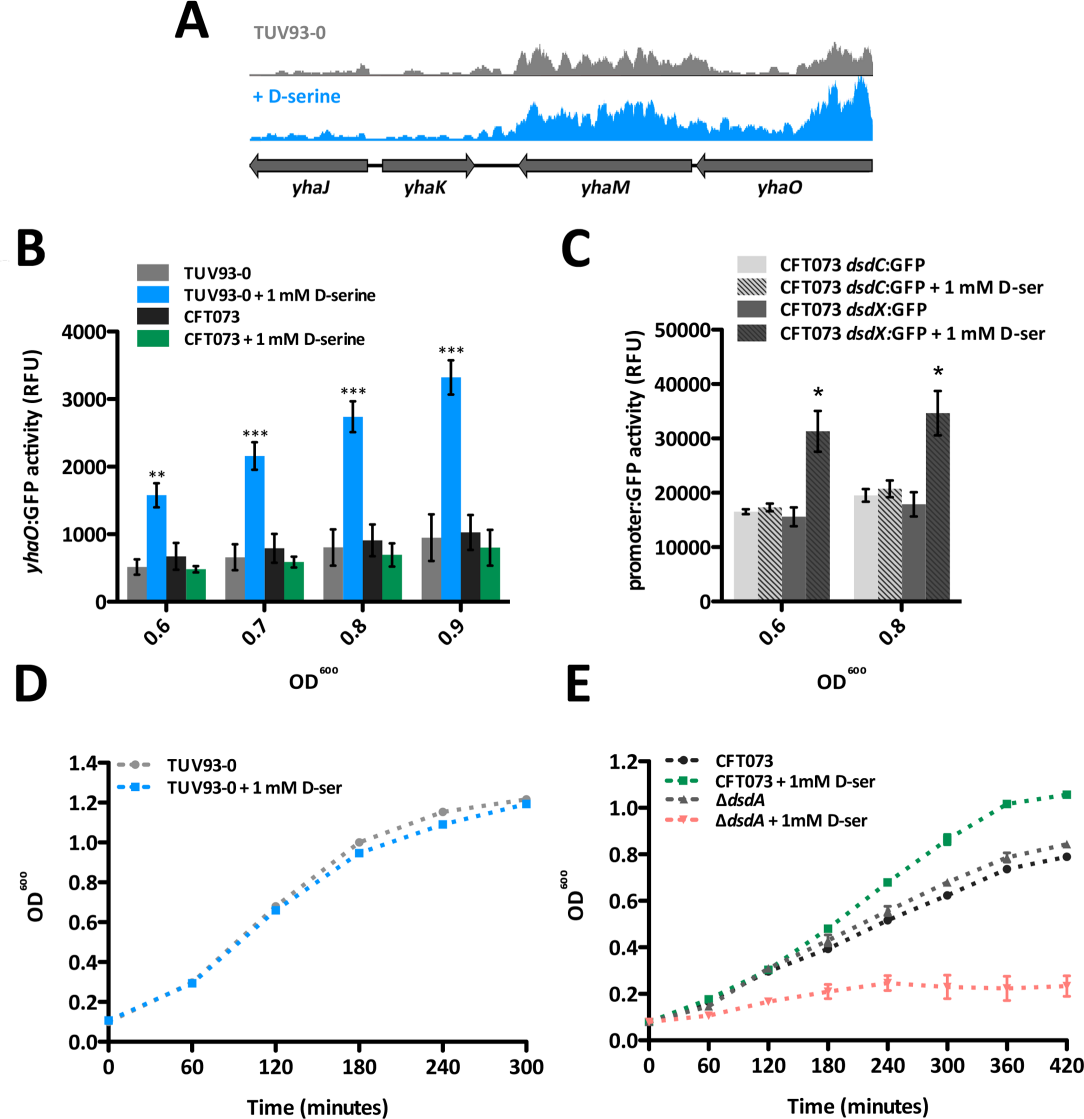
Transcriptional regulation of D-serine uptake in EHEC and UPEC. (A) Coverage of RNA-seq reads across the *yhaOMKJ* gene cluster. Data was mapped from previously obtained RNA-seq data that investigated the response of TUV93-0 gene expression to D-serine [21]. The height of coverage peaks has been scaled to that of TUV93-0 using EasyFig [35]. TUV93-0 transcript expression is highlighted in grey whereas TUV93-0 plus D-serine is highlighted in blue. (B) *yhaO* expression is responsive to environmental D-serine. TUV93-0 and CFT073 were transformed with a plasmid containing a GFP-*yhaO* promoter fusion (p*yhaO*:GFP). Activity of the *yhaO* promoter was measured during growth in relative fluorescence units (RFU) using TUV93-0 alone in MEM-HEPES (grey), TUV93-0 supplemented with 1 mM D-serine (blue) and CFT073 alone (dark grey) or supplemented with 1 mM D-serine (green). Data was calculated from three biological replicates and plotted at increasing OD^600^ values. ** and *** denote P ≤ 0.01 and P ≤ 0.001 respectively. (C) *dsdC* and *dsdX* expression in response to D-serine in UPEC. CFT073 was transformed with p*dsdC*:GFP (light grey) and p*dsdX*:GFP (dark grey) transcriptional reporters and activity of measured during growth in MEM-HEPES with and without 1 mM D-serine (clear or dashed bars respectively) as relative fluorescence units (RFU). Data was calculated from three biological replicates. * denotes P ≤ 0.05. (D) Growth curves of EHEC TUV93-0 in MEM-HEPES alone (grey) and supplemented with 1 mM D-serine (blue). (E) Growth curves of UPEC CFT073 and UPEC *ΔdsdA* in MEM-HEPES alone (black, grey) and supplemented with 1 mM D-serine (green, red). Growth experiments were performed in biological triplicate.

Examination of previous RNA-Seq data revealed that transcripts mapped to the co-transcribed *yhaO* and *yhaM* were increased (~1.5-fold) in the presence of D-serine when compared to TUV93-0 alone in MEM-HEPES (Fig 6A), whereas the transcription of *yhaJ* was not affected by addition of D-serine. To further explore the regulation of YhaO, a reporter plasmid containing the promoter region of *yhaO* fused to GFP in-frame (p*yhaO*:GFP) was generated and transformed into TUV93-0. Expression of GFP was determined during growth in MEM-HEPES supplemented with 1 mM D-serine. Addition of D-serine resulted in a significant increase in GFP production during mid to late exponential phase of growth, with expression increasing over this growth phase from a 3.1-fold increase (OD^600^ of 0.6) to a 3.5-fold increase above that of the wild type alone (OD^600^ of 0.9). In contrast, UPEC CFT073 transformed with the p*yhaO*:GFP reporter displayed similar level of *yhaO* expression in the absence of D-serine but no increase in GFP production when supplemented with 1 mM D-serine under these conditions, regardless of the growth phase (Fig 6B). The expression of *yhaJ* and *yhaO* in EHEC is comparable to that of *dsdC* and *dsdX* in UPEC, in that D-serine does not affect transcription of the regulator *dsdC* but the transporter *dsdX* is significantly upregulated by its presence in the growth media. This was examined using CFT073 transformed with reporters containing the *dsdC* and *dsdX* promoters fused to GFP, revealing a 2-fold increase in *dsdX* expression when D-serine was present (Fig 6C). Growth of EHEC under these conditions was not affected by the addition of D-serine (doubling rates of 49.8 and 51.2 minutes respectively), despite an increase in its uptake from the environment (Fig 6D). Contrastingly, wild type UPEC displayed a growth advantage in the presence of D-serine (doubling rates of 150.2 and 103.2 minutes for without and with D-serine respectively; P-value 0.0023) but a *ΔdsdA* mutant was drastically impaired for growth in the presence of D-serine (doubling rates of 142.5 and 221.4 minutes respectively; P-value 0.0127) (Fig 6E). Collectively these results suggest that EHEC has an unusual tolerance for D-serine in the environment and are consistent with the notion that YhaO is an L/D-serine transporter but demonstrate that regulation of *yhaO* is responsive to the presence of environmental D-serine in an EHEC background only.

### YhaJ is involved in the regulation of *yhaO* in EHEC

**Fig 7:**
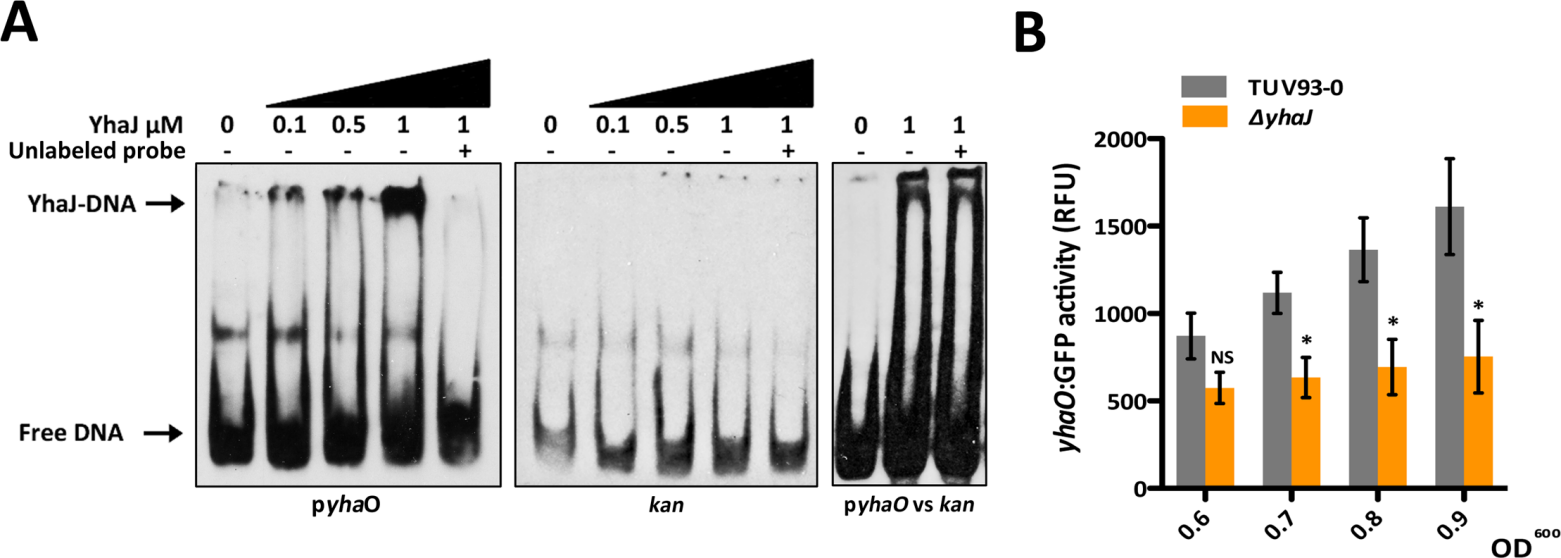
YhaJ directly regulates *yhaO* expression in EHEC. (A) Purified YhaJ was tested for its ability to bind the *yhaO* promoter region (*pyhaO;* ~300 bp region upstream of the *yhaO* coding sequence) by EMSA. DIG-labeled *pyhaO* was incubated with increasing concentrations of YhaJ that corresponded to a shift in free-DNA indicating a YhaJ-DNA complex. Specificity of the binding reaction was tested by the addition of a 100-fold excess (+) of unlabeled *pyhaO* probe to the binding reaction to outcompete binding of the DIG-labeled probe to YhaJ. These reaction conditions were carried out using a fragment of the *kan* gene as a negative control. Additionally, the unlabeled *kan* probe in 100-fold excess was used as a non-specific competitor for YhaJ binding to the DIG-labeled *yhaO* probe (p*yhaO* vs *kan*). (B) Activity of the *yhaO* promoter in the *ΔyhaJ* mutant background. A plasmid containing a GFP-*yhaO* promoter fusion was transformed into TUV93-0 and *ΔyhaJ* to monitor transcription of *yhaO* in RFU during growth in MEM-HEPES. Data was calculated from three biological replicates and plotted at increasing OD^600^ values. * denotes P ≤ 0.05.

Having explored the function of YhaO, we aimed to better understand the role of YhaJ. Given the protein has strong homology to known LTTRs, we investigated if YhaJ indeed functioned as a DNA-binding protein and how it contributed to the regulation of gene expression in EHEC. The gene encoding YhaJ from EDL933 was cloned into pET28b, over-expressed in *E*. *coli* BL21 (DE3) cells and purified using a combination of immobilized nickel affinity chromatography and size exclusion chromatography. LTTRs typically form dimers in solution that can interact as dimer pairs to bind DNA specifically and regulate gene expression either positively or negatively [36]. Given the genetic location of *yhaJ* in respect to *yhaO* as described above we hypothesized that YhaJ may regulate *yhaO* expression by interacting with its upstream promoter region. Electrophoretic mobility shift assay (EMSA) analysis was carried out using a DIG-labeled DNA probe corresponding to an ~300 bp region upstream of the *yhaO* ATG start codon. Incubation of this probe with increasing concentrations of purified YhaJ (0.1 to 1 μM) resulted in a shift of the free DNA indicative of a protein-DNA complex (Fig 7A). In order to address the specificity of this reaction we employed three approaches. First, 1 μM YhaJ was incubated with the DIG-labeled *yhaO* probe and a 100-fold excess of unlabeled *yhaO* probe. The unlabeled probe outcompeted the DIG-labeled probe resulting in loss of band shift pattern. Second, YhaJ was tested for its ability to bind a fragment of the *kan* gene as a negative control. Incubation of increasing concentrations of YhaJ with DIG-labeled *kan* probe induced no band shift as seen for the *yhaO* probe. Third, a 100-fold excess of unlabeled *kan* probe used as a non-specific competitor for the DIG-labeled *yhaO* probe in a reaction with 1 μM YhaJ could not inhibit binding. These results collectively indicate that YhaJ can specifically bind the upstream regulatory region of *yhaO* (Fig 7A). Having established this, we next investigated the role YhaJ plays on *yhaO* transcription. TUV93-0 and the *ΔyhaJ* mutant were transformed with p*yhaO*:GFP and were cultured in MEM-HEPES to promote expression of *yhaO* under LEE-inducing conditions. Activity of the *yhaO* reporter increased steadily into the late exponential phase in TUV93-0, conditions that promote increased LEE expression, but was significantly (P ≤ 0.05) impaired in *ΔyhaJ* (Fig 7B). *yhaO* expression was reduced 1.5-fold from that of the wild type at OD^600^ of 0.6 but this increased to a >2-fold at OD^600^ 0.9. Together these data suggest that YhaJ plays a part in regulating *yhaO* expression directly under conditions that promote expression of the LEE.

### YhaJ directly activates *LEE1* transcription in EHEC

**Fig 8:**
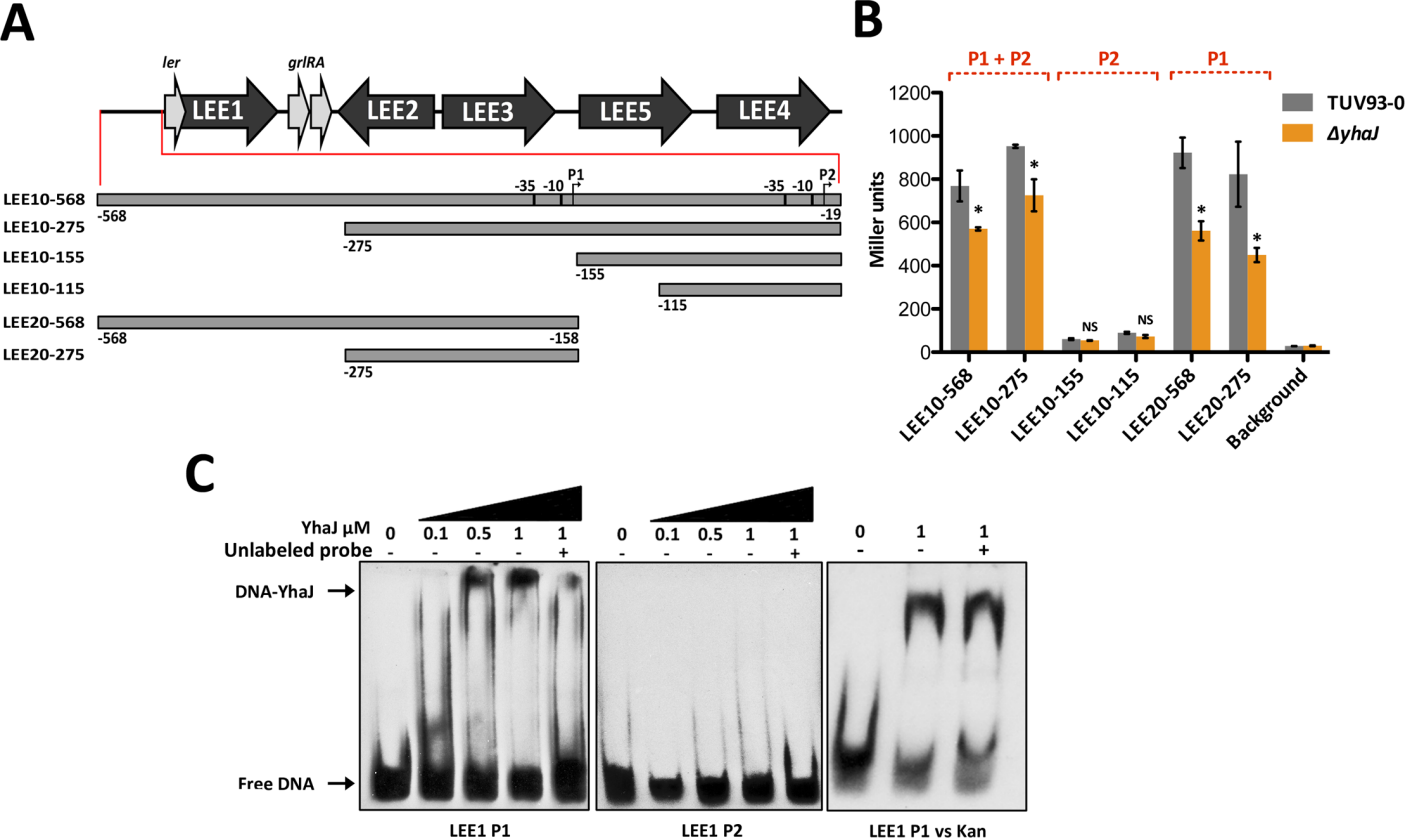
YhaJ directly regulates the LEE in EHEC. (A) Schematic representation of the LEE pathogenicity island. The master regulator *ler* upstream regulatory region is expanded to illustrate the rationale behind the design of the nested deletion series to monitor *LEE1* promoter activity as described by Islam *et al*. Promoters P1 and P2 as well as corresponding -10 and -35 elements are indicated. (B) Monitoring the impact of YhaJ on *LEE1* expression in TUV93-0. LEE10 and LEE20 plasmids were transformed into TUV93-0 (grey) and *ΔyhaJ* (orange) and LacZ activity was measured in Miller units at an OD^600^ of approximately 0.7 during growth in MEM-HEPES. The presence of promoters P1 and P2 in each assay is indicated above the graph. * and NS denote P ≤ 0.05 and no significance respectively and the data was calculated from three biological replicates. (C) Purified YhaJ was tested for its ability to bind the *LEE1* P1 and P2 promoter regions by EMSA. DIG-labeled LEE1 P1 and P2 specific DNA probes were incubated with increasing concentrations of YhaJ. A shift in free-DNA that corresponds to a YhaJ-DNA complex was only observed for *LEE1* P1 and this was in agreement with the data presented in panel B. Specificity of the binding reaction was tested by the addition of a 100-fold excess (+) of unlabeled P1 or P2 probe to the binding reaction to outcompete binding of the DIG-labeled probe to YhaJ. A 100-fold excess of unlabeled *kan* probe was also used as a non-specific competitor for YhaJ binding to the P1 region (*LEE1* P1 vs *kan*) to ensure specify of the band shift pattern. EMSA experiments were performed in triplicate to confirm the results.

Given the importance of YhaJ for virulence, we hypothesized that this transcriptional regulator may also control the LEE directly. To address this, we utilized a set of previously described reporters that contain nested deletions of the *LEE1* regulatory region fused to *lacZ* encoding the beta-galactosidase gene [37]. These reporters are designed to monitor activity of the *LEE1* regulatory region from both its P1 (distal) and P2 (proximal) promoters, which have both been documented to play distinct roles in *LEE1* activation [37–39]. A schematic of the reporter system is illustrated in Fig 8A and includes LEE10-568/LEE10-275 (P1 and P2 containing), LEE10-155/LEE10-115 (P1 containing) and LEE20-568/LEE20-275 (P2 containing). Monitoring the activity of this system in both TUV93-0 and *ΔyhaJ* under LEE-inducing conditions revealed that YhaJ regulated *LEE1* transcription at the major P1 promoter (Fig 8B). *ΔyhaJ* showed significantly (P ≤ 0.05) reduced expression of *LEE1* activity in LEE10-568/LEE10-275 and LEE20-568/LEE20-275 but not in the promoter P2-only LEE10-155/LEE10-115 constructs (Fig 8B). Moreover, we used EMSA analysis to determine the binding capacity of YhaJ to the P1 and P2 promoter regions. In agreement with the transcriptional reporter data, increasing concentrations of YhaJ incubated with a DIG-labeled P1 probe induced a band shift that could be outcompeted by a 100-fold excess of unlabeled P1 probe. Conversely, YhaJ was not able to bind the P2 promoter region indicating specificity of YhaJ to the *LEE1* P1 promoter region. As an additional control, a 100-fold excess of unlabeled *kan* probe was unable to reverse binding of YhaJ to the P1 promoter region (Fig 8C). Interestingly, despite deletion of *yhaJ* being detrimental to NLE transcription as seen in the RNA-seq data, purified YhaJ showed no binding capacity to a selection of DIG-labeled probes corresponding to distinct NLE promoter regions (S7 Fig). NLE regulation is less understood than that of the LEE and not all NLEs are not under the control of a universal regulatory system but have been shown to be influenced by LEE encoded regulators Ler and GrlA [28,40,41].

Taken together, these data strongly suggest that YhaJ plays an important role in directly activating *LEE1* transcription and thus the entire LEE PAI via Ler. These results explain why deletion of *yhaJ* has detrimental effects on the virulence potential of TUV93-0. Given our previous work investigating the ability of EHEC to respond to environmental D-serine by downregulating the LEE, these results also offer mechanistic reasoning behind the regulation of the D-serine transporter *yhaO* by YhaJ.

## Discussion

The ability of a pathogen to sense and respond to stimuli presented in the environment is of critical importance for niche adaptation. Intestinal pathogens must not only be able to identify their preferred site of colonization in terms of nutrient availability but also must compete with the resident microbiota for limited nutrients. Colonization within this complex ecosystem therefore requires effective sensing systems to ensure appropriate gene expression. There has been an emergence in the literature of a wide variety of important signals that EHEC can encounter in the intestinal tract and the complex molecular basis behind how these signals are interpreted allowing colonization of a particular niche within the host gastrointestinal tract is beginning to be unraveled in detail [9].

The precise signals that may contribute to determining niche selection extraintestinally are a less explored area. We recently described how the host metabolite D-serine selectively downregulated the LEE-encoded T3SS by modulating the expression of pre-existing transcriptional regulators [21]. D-serine is found in abundance at extraintestinal sites such as the brain and urinary tract but its concentrations along the intestine are below that required to inhibit LEE expression or growth of EHEC. Conversely, D-serine acts as a positive fitness trait and regulator of virulence gene expression in UPEC and the Gram-positive urinary tract pathogen *Staphylococcus saprophyticus* [22,24,42,43]. We also analysed the genome sequences of 1581 *E*. *coli* isolates and found that carriage of both the *dsdCXA* D-serine tolerance locus and the LEE PAI was a significantly rare event. By introducing a functional DsdA deaminase to EHEC the ability to use D-serine as a carbon source and eliminate intracellular accumulation was recovered however inhibition of the LEE was not abolished. This led us to propose that there was an ‘evolutionary incompatibility’ between the two loci thus assisting in the restriction of LEE-positive pathogens to the intestinal tract irrespective of whether they could catabolize D-serine or not [21]. This hypothesis offers one possible explanation as to why extraintestinal pathogens do not carry and adapt the LEE-encoded T3SS to their advantage, despite the LEE T3SS facilitating binding, at least *in vitro*, to a wide variety of cell types [41,44,45]. Acquisition of the LEE is also not determined by the phylogenetic relatedness of *E*. *coli* strains. Certain strains of intestinal pathogenic EPEC for instance are more closely related to ExPEC members of the B2 phylogroup yet have acquired the LEE, lost *dsdCXA* and consequently have evolved to be dedicated intestinal pathogens.

Despite this exciting finding the underlining molecular mechanism used to mediate D-serine repression of the LEE remained a key question. Classically, bacteria utilise two-component sensors to respond to many changes in the environment and regulate gene expression. EHEC use the FusKR two-component system and quorum sensing systems respectively to respond to fucose concentrations appropriately at the epithelial surface and to integrate host and bacteria derived hormone-like signals [12,46]. Other signals such as ethanolamine via its sensing regulator EutR can be sensed directly within the cell to modulate the activity of virulence associated transcriptional regulators, [13,47]. For the latter, bacterial cells must be able to uptake the required signal in order to respond to it at the cytoplasmic level.

While possessing DsdX as a pseudogene, EHEC accumulate D-serine intracellularly implying other uptake systems are at play. In this study we have identified YhaO, an inner membrane transporter that is capable of transporting D-serine in EHEC. This gene is part of a cluster that includes the co-transcribed YhaM (a D-serine dehydratase) and YhaJ (a DNA-binding LTTR). Despite functionality of YhaO being confirmed, YhaM is clearly non-functional in EHEC and could not restore a UPEC *dsdA* mutant when supplied *in trans*. YhaJ was found to be responsible, at least in part, for regulating *yhaO* expression particularly at late exponential phase of growth. This is interesting as when EHEC is grown in MEM-HEPES, LEE expression is dramatically increased at this stage. Previous microarray data identified increased *yhaO* expression under these conditions also and recently an independent study reported *yhaO* to be part of the wider Ler regulon in EPEC but no role was specified [28,48]. Deletion of *yhaO* was detrimental for LEE expression and therefore A/E lesion formation. Together, these data imply an important role for YhaO in LEE regulation specifically under inducing conditions.

**Fig 9:**
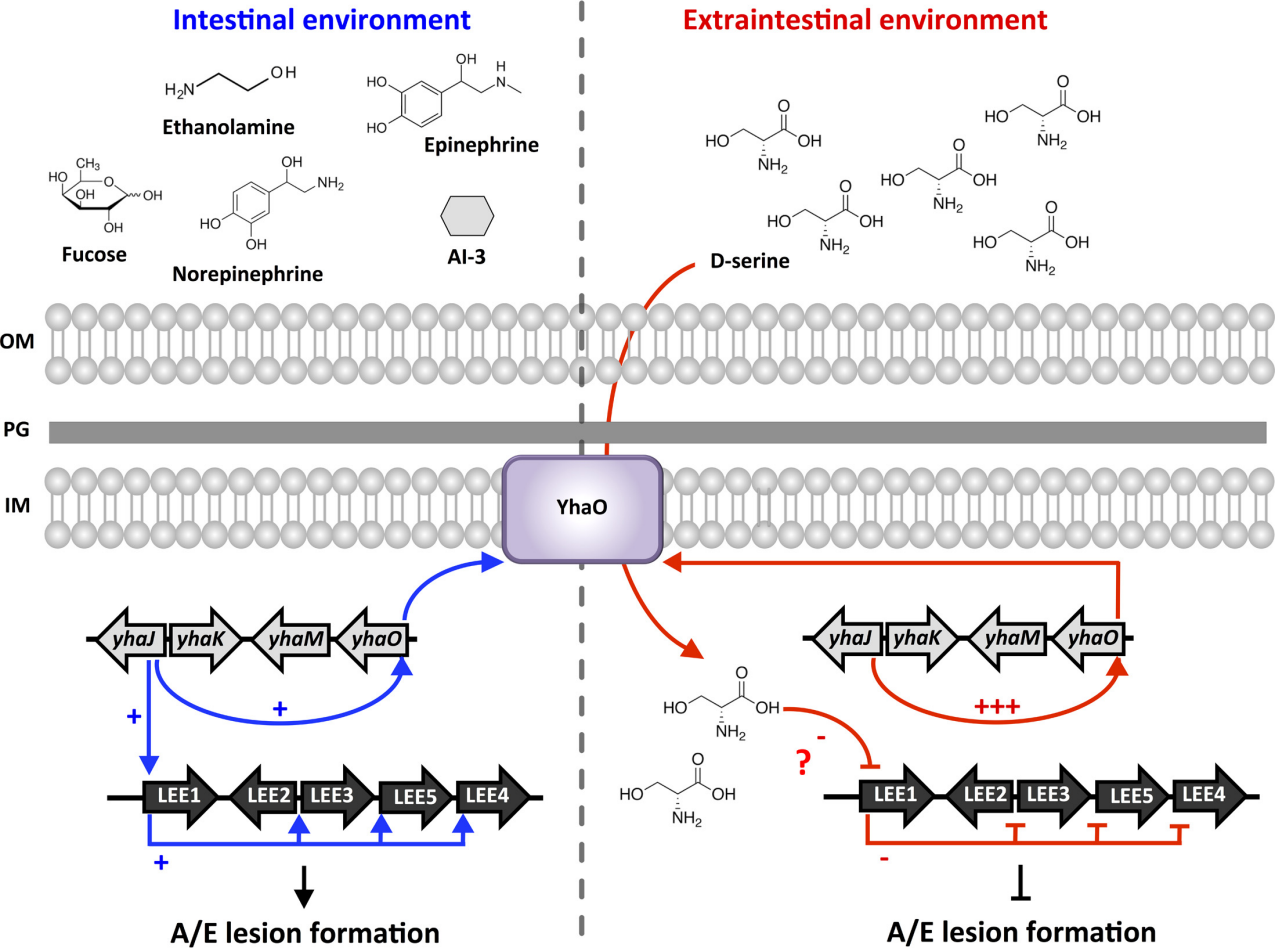
Schematic model of LEE regulation by the YhaO/YhaJ D-serine sensory system. Summary of small molecule signals that are encountered by EHEC in the intestinal (red) and extraintestinal (blue) environments [9]. In the intestinal environment LEE expression is affected by signals such as fucose, ethanolamine and quorum sensing molecules (epinephrine, norepinephrine and AI-3). YhaJ constitutively regulates *yhaO* as well as stimulating the LEE (+) helping to promote A/E lesion formation and colonization of host tissue. In the extraintestinal environment D-serine can be encountered in high concentrations leading to repression (-) of the LEE by an unknown (?) direct mechanism [21]. Expression of *yhaO* is also increased resulting in further uptake of D-serine and thus a greater transcriptional response to this signal (+++) promoting inhibition of colonization in unfavorable environments. The outer membrane (OM), peptidoglycan layer (PG) and inner membrane (IM) of EHEC are indicated.

Expression of *yhaO* was found to be responsive to exogenous D-serine in EHEC, increasing largely in its presence, but *yhaJ* did not express differentially. This is a similar scenario to that of the D-serine tolerance locus in UPEC in that *dsdC* transcription is not increased in response to D-serine but *dsdX* is significantly upregulated. This seems counter-productive considering the inhibitory effects of D-serine reported for *E*. *coli* [49]. Surprisingly, EHEC is able to tolerate mM concentrations of D-serine in MEM-HEPES without any growth defect, a trait that a UPEC *ΔdsdA* mutant does not have. We recently reported that wild type EHEC and UPEC *ΔdsdA* accumulate D-serine intracellularly and that this is due to the lack of a functional DsdA [21]. We also found the repressive effects of D-serine on LEE transcription to be a concentration-dependent process. In this respect, increasing D-serine uptake in EHEC would therefore increase the transcriptional response to this amino acid, all the while still allowing tolerance to intracellular accumulation. This provides a logical role for expression of YhaO under LEE-inducing conditions, to act as a safety net constantly monitoring the environment for inhibitory concentrations of D-serine. Further evidence for this is in the fact that YhaJ is expressed at a constant level providing continued input into the *yhaO* promoter. Constitutive expression of *yhaJ* also benefits expression of the LEE. We found YhaJ to be necessary for full expression of LEE operons 1 through 5 and formation of A/E lesions on host cells. Furthermore, YhaJ was found to be a direct regulator of the *LEE1* P1 promoter in EHEC, which is considered the major activator of *ler* expression [37]. By feeding directly into the LEE master regulator and also regulating YhaO, YhaJ provides unique and subtle control over colonization in EHEC (Fig 9).

Pathogen emergence is not solely influenced by the acquisition of virulence factors. Horizontally acquired elements such as the LEE must be integrated into the regulatory network of the cell in order to function in a timely and appropriate manner. Regulators of the LEE are often acquired horizontally on mobile genetic elements. For instance, the Pch regulators found on cryptic-prophage and the prophage-encoded Psr regulators that usurp the conserved glutamate dependent (GAD) acid stress response to regulate the LEE indirectly [50,51]. However, chromosomally encoded global regulators, such as nucleoid-associated proteins, are also important regulators of virulence [52–55]. Either way, the LEE is not a ubiquitous system and receives regulatory inputs from diverse sources. This demonstrates the high degree of adaptability in the *E*. *coli* genome. The same can be said of UPEC isolates. UPEC harbor a vast array of virulence determinants that can vary between isolates but no one virulence factor is responsible for colonization of the urinary tract. That being said it has been documented previously that certain UPEC isolates mutated for the *dsdCXA* D-serine tolerance locus have reacquired these genes horizontally presumably due to their importance for UPEC fitness and virulence in the urinary tract [56]. A recent study documenting host-specific induction of fitness genes during human urinary tract infection also highlighted the importance of typically non-virulence associated mechanisms in the pathogenesis of UPEC [57]. UPEC is very capable of surviving in the gastrointestinal tract in a highly competitive manner yet, despite carrying virulence factors that promote dissemination extraintestinally, UPEC is a very adapted pathogen tailoring the transcriptional profile of its core genome specifically to its current environment [58]. EHEC has the potential to at least come into contact with the urinary tract upon exit from the host via the rectum and in some rare cases has even been isolated from hospitalized patients suffering from urinary tract infection [59]. However, these variants are not clinically prevalent and this environment does not offer much advantage to the intestinally adapted EHEC. Intriguingly, Subashchandrabose *et al*. identified *yhaOMKJ* as being differentially expressed in a selection of UPEC isolates from human urinary tract infection when transcription *in vivo* was compared with growth in LB broth or human urine. This suggests an as yet unidentified role for these genes in UPEC during infection of the bladder, a D-serine rich environment [57]. This observation supports our model that YhaO and YhaJ play important roles in gene regulation during colonization and that they help shape the specialization process of pathogenic *E*. *coli*. Therefore, it is perfectly plausible that EHEC must possess a system to sense abundant signals including those from the urinary tract such as D-serine, thus informing the pathogen to restrict the expression of its key colonization factor when approaching a new, less favorable environment.

Adaptation of existing core genes to ensure appropriate regulation of horizontally acquired elements is important for maximizing bacterial competitiveness. Recent work has shown that in the mouse pathogen *Citrobacter rodentium*, which also utilizes a LEE-encoded T3SS for virulence, an AraC-like regulator RegA responds to bicarbonate ions to regulate transcription globally, including the LEE. RegA homologues are found in *E*. *coli* species but do not play a role in LEE regulation, despite being of similar evolutionary origin, further demonstrating pathogen specific regulatory adaptations to common virulence factors [60–62]. The *yhaOMKJ* locus is similarly very conserved across the *E*. *coli* phylogenetic spectrum. With maintenance being intact among the vast majority of *E*. *coli*, this implies a role that is important for the fitness of both pathogens and non-pathogens in a range of different hosts and environments. RNA-seq data described here provide evidence for this, as YhaJ and YhaO seem to be involved in global gene regulation suggesting other roles besides control of the LEE. In contrast, the minority of *E*. *coli* that show attrition in this locus–particularly *Shigella* for *yhaJ*–are, presumably, undergoing a ‘use it or lose it’ approach to niche specification and genome minimalism [63]. Clearly in EHEC, YhaO and YhaJ are important for full expression of the LEE, a specialized colonization factor that is absent from non-pathogens. However, in over half of the *Shigella* genomes analysed, *yhaJ* was present as a pseudogene. As an invasive pathogen YhaJ may not be essential for other processes that it may regulate, including the control of specialized virulence factors. Additionally, it is possible that exposure to D-serine is a less common occurrence for *Shigella*, and therefore a role for YhaJ is less important resulting in attrition of the gene [64]. Certainly this observation is consistent with our previous findings suggesting that host metabolism can drive bacterial evolution by providing selective environments. Notably, *Shigella* carry a plasmid-encoded T3SS that is genetically related to the ETT2 (*E*. *coli* type III secretion 2) system also carried by MNEC isolates [65]. Distinct from the LEE in both genetics and regulation, we predict the ETT2 would not be downregulated by addition of D-serine and we are currently testing this postulate.

Overall, we have identified a highly conserved and widely distributed transporter and transcriptional regulator that act in concert to sense D-serine and control gene expression. Based on these findings, we propose to name YhaO as DlsT (D/L-serine transport protein). The precise role of YhaJ in global gene regulation is not yet fully determined; hence we choose not to suggest a functional name as this time. We postulate this ubiquitous system is important as D-serine can act as either a carbon source or stress depending on the genetic background of the individual pathotype and modulate gene expression of unique virulence factors. Irrespective of its nutritional value, it appears that the ability to effectively monitor and respond to D-serine in the environment is critical for maximizing bacterial fitness and niche specification in certain pathotypes. This study demonstrates the adaptive power of the existing *E*. *coli* core genome to recycle and reuse present genes for a more specialized function.

## Materials and Methods

### Bacterial strains, plasmids, primers and growth conditions

The bacterial strains, plasmids and oligonucleotide primers used in this study along with relevant accompanying information are listed in S3, S4 and S5 Tables, respectively. Single bacterial colonies were inoculated into 5 ml LB broth containing the appropriate antibiotics and cultured overnight at 37°C, 200 rpm. Overnight cultures were used to inoculate pre-warmed MEM-HEPES (Sigma, St Louis, MO, USA; cat # m7278) and samples were cultured at 37°C, 200 rpm. D-serine was purchased from Sigma. Motility was assessed by inoculating the center of on 0.25% Tryptone agar plate with 5 μl of bacterial culture at OD^600^ 0.6 and diameter of the population swim was measured after 8 hours at 31°C.

### Generation of *yhaO*, *yhaM*, *yhaK* and *yhaJ* deletion strains

Non-polar mutations of *yhaO*, *yhaM*, *yhaK* and *yhaJ* were generated in TUV93-0. *ΔyhaO* was generated using Lambda red-mediated mutagenesis [66]. *ΔyhaM*, *ΔyhaK* and *ΔyhaJ* were generated using allelic exchange [67]. Complementation was achieved by cloning *yhaJ* and *yhaO* into low copy number pWSK vectors and transforming the plasmids into each deletion background.

### *In vitro* GFP reporter-fusion assay of promoter activity

Strains of interest were transformed with p*yhaO*:GFP, p*dsdC*:GFP or p*dsdX*:GFP [68] and used to inoculate 10 ml MEM-HEPES as described above. Replicates were cultured at 37°C and 200 rpm with samples taken every hour for measurement of bacterial density (OD^600^) and fluorescence. Population GFP expression was determined by transferring 200 μl aliquots to a black 96-well plate for GFP fluorescence measurement (excitation at 485 nm; emission at 550 nm) on a FLUOstar Optima Fluorescence Plate Reader (BMG Labtech, UK). GraphPad Prism 5.0 (San Diego, CA, USA) software was used to generate a standard curve of OD^600^ versus fluorescence and obtain values at specific OD^600^ for comparison between samples. Background fluorescence was corrected for by subtracting values gained from cells carrying promoter-less reporter plasmids from a standard curve. Data presented are the mean (±SEM) of at least three biological replicates.

### Beta galactosidase assay of *LEE1* promoter activities

The nested deletion series of the *LEE1* promoter region fused to *lacZ* were generated and described elsewhere [37]. These constructs were transformed into wild type TUV93-0 and *ΔyhaJ*. Promoter activity was measured as described previously [69]. Briefly, transformed strains were cultured in MEM-HEPES as described above to an OD^600^ of ~0.8. 400 μl aliquots were diluted 1:1 in Z buffer and lysed with 0.1% SDS and chloroform then vortexed before incubating at 37°C for 1 minute. 200 μl of o-nitrophenyl-β-d-galactopyranoside (2 mg/ml) was added to each reaction and incubated at 37°C before stopping the reaction with 500 μl of 1 M Na_2_CO_3_ when sufficient color change was observed. OD^450^ was measured and used to calculate the promoter activity in Miller units. Data presented are the mean (±SEM) of at least three biological replicates.

### SDS-PAGE and immunoblot analysis of Type III secreted proteins

SDS-PAGE analysis of secreted proteins was carried out as described previously [51]. Strains of interest were cultured in 50 ml MEM-HEPES as above to an OD^600^ of ~0.8 and supernatants were obtained by centrifugation at 4000 rpm for 20 minutes. Whole cell pellets were lysed using BugBuster Protein Extraction buffer (Merck, New Jersey, USA). For secreted proteins, cell culture supernatants were syringe filtered (0.45 μm) and precipitated with 10% v/v TCA (Sigma) overnight at 4°C. Secreted proteins were harvested by centrifugation at 4000 rpm (4°C) for 1 hour. Protein pellets were resuspended in Tris-HCl (pH 8.0) and analyzed by SDS-PAGE using the Novex system (Invitrogen, Carlsbad, CA, USA). Primary antibodies used for immunoblotting were EspD (1/6000), Tir (1/2000) and GroEL (1/20000). Antibodies were made up in PBST containing 1% skim milk powder. Comparison of protein levels from SDS PAGE and immunoblot experiments was carried out by densitometry using ImageJ. Experiments were performed on multiple occasions to confirm the results.

### Total RNA extraction and mRNA enrichment

Bacterial cultures were grown as above and mixed with two volumes of RNAprotect reagent (Qiagen, Valencia, CA, USA), incubated for 5 minutes at room temperature and cell pellets harvested by centrifugation. Total RNA was extracted using an RNeasy kit (Qiagen) after which genomic DNA was removed using TURBO DNase (Ambion, Carlsbad, CA, USA). Total RNA samples were enriched for mRNA using MICROBexpress mRNA enrichment kit (Ambion). Samples for RNA-seq analysis were quality control tested for mRNA enrichment using an Agilent Bioanalyzer 2100 at the University of Glasgow, Polyomics Facility.

### RNA-seq transcriptome analysis

cDNA synthesis and sequencing was performed at the University of Glasgow Polyomics Facility (Illumina NextSeq 500) and the Edinburgh Genomics facility (Illumina HiSeq 2500) for the *ΔyhaJ and ΔyhaO* mutants respectively obtaining 75 or 100 bp single end reads. Samples were sequenced in triplicate with TUV93-0 replicates sequenced in parallel on each platform. Raw reads were QC checked using FastQC (Babraham Bioinformatics, Cambridge, UK) and trimmed accordingly using CLC Genomics Workbench (CLC Bio, Aarhus, Denmark). Trimmed reads were mapped to the EDL933 reference genome (NCBI accession number: NC_002655.2) allowing for 3 mismatches per read and at least 5 reads per feature. Analysis of differential expression was performed using the Empirical analysis of DGE tool, which implements the EdgeR Bioconductor tool [70]. Differentially expressed genes were identified using a positive or negative absolute fold change of ≥1.5 and a corrected P-value of ≤ 0.05 (false-discovery rate of 5%). GO functional grouping was summarized according to information available on Colibase and the RegulonDB [71,72]. Volcano plots were generated in CLC Genomics Workbench. Coverage plots used to visualize the pattern and abundance of RNA-seq read mapping to genomic loci were generated using EasyFig [35]. The sequence reads in this paper have been deposited in the European Nucleotide Archive under study PRJEB12065.

### Quantitative real time PCR (qRT-PCR)

Validation of RNA-seq data was carried out by qRT-PCR using KAPA SYBR FAST Universal qRT-PCR master mix (KAPA Biosystems, Woburn, MA, USA) and M-MLV Reverse Transcriptase (Promega, Madison, WI, USA). Total RNA was extracted as described above and was quantified on a NanoDrop 2000 (Thermo-Scientific). Samples for comparison were normalized to a concentration of 50 ng/μl using TE buffer (Ambion, Carlsbad, CA, USA). qRT-PCR analysis was performed using a one-step reaction; cDNA synthesis first followed by qRT-PCR according to the manufacturers specifications. Individual reactions were performed in technical triplicate and each gene to be analysed was performed in biological triplicate. The *gapA* gene was used to normalize the results. qRT-PCR reactions were carried out using the ECO Real-Time PCR System (Illumina, San Diego, CA, USA) according to the manufacturers specifications and the data were analysed according to the 2^-ΔΔCT^ method [73].

### Host cell adhesion assay and fluorescence microscopy

Cell adhesion assays were performed essentially as described previously [21]. Coverslips were seeded with 4x10^4^ human epithelial tissue culture cells (HeLa cells; Thermo-Scientific, Waltham, MA, USA) in multi-well plates and incubated overnight in MEM-HEPES at 37°C with 5% CO2. TUV93-0, *ΔyhaJ and ΔyhaO* were transformed with an RFP-expressing plasmid (pRFP) and subsequently used for adhesion assays. Cultures for cell infection were grown in MEM-HEPES at 37°C until at an OD^600^ of ~0.7. HeLa cells were washed with fresh MEM-HEPES and infected with 100 μl bacterial culture (OD^600^ of 0.1) in 500 μl fresh pre-warmed MEM-HEPES. Multi-well plates were centrifuged at 400 rpm for 3 minutes and incubated at 37°C with 5% CO_2_ for 2 hours, after which coverslips were washed with fresh media to remove unbound bacteria and incubated for a further 3 hours. Coverslips were washed 3 times with sterile PBS before fixing for 20 minutes with 250 μl PFA (2%). Coverslips were washed a further 3 times with PBS. 250 μl of Triton X-100 (0.5%) was added and wells were incubated for 5 minutes before further washing. Host cell actin was stained with Phalloidin-488 (Invitrogen) for 1 hour and coverslips were washed again. Mutants carrying complementation plasmids were stained at this stage with anti-O157 primary (1/500) and Alexa-Fluor 555 secondary (Invitrogen) as an alternative to expression of pRFP. Finally, coverslips were wicked dry and mounted on glass microscope slides using fluorescent mounting medium (Dako, Cambridge, UK). Slides were dried in the dark overnight before imaging. Imaging was performed on a Zeiss M1 Axioimager microscope and data was acquired and deconvoluted using the Zen Pro software (Zeiss, Jena, Germany). For quantification of adhesion at least twenty-five random fields of view were obtained per replicate and A/E lesions were counted as areas of intense actin condensation beneath bacterial cells. Data presented are the mean (±SEM) of at least three biological replicates.

### Growth on D-serine as a sole carbon source

Assessment of the ability to grow using D-serine as a sole carbon source was carried out as described previously using MOPS minimal media agar plates supplemented with D-serine [23]. Strains of interest were streaked and grown over a 36-hour period at 37°C on MOPS plates. Experiments were performed multiple times to confirm the results.

### Recombinant YhaJ purification

The coding sequence for *yhaJ* from EDL933 was amplified from TUV93-0 genomic DNA by PCR using primers with BamHI (forward) and HindIII (reverse) in-frame flanks and cloned into pET-28b (N-terminal 6xHistidine tag) before being transformed into *E*. *coli* BL21 DE3 cells for over expression. Overnight cultures were used to inoculate LB and cultures were grown at 37°C and 200 rpm until OD^600^ of 0.6, at which point cells were induced with 1 mM IPTG and overexpressed at 15°C overnight. Cells were harvested by centrifugation, resuspended in wash buffer (200 mM NaCl, 50 mM Tris, 40 mM Imidazole, 10% glycerol) and lysed using a French press. The supernatant was then used for immobilized metal affinity ion chromatography (IMAC) using an AKTA-prime according to the manufacturers specifications. Further purification of desired elution samples was carried out using size-exclusion chromatography (SEC). Elution fractions from the IMAC stage were dialyzed overnight in SEC buffer before being re-purified according to size using an AKTA-prime and a Superdex S200 column. Elution fractions corresponding to the size of YhaJ were retained. Concentration of purified YhaJ was determined using a Nanodrop 2000.

### Radiolabelled D-serine transport assay

In order to examine the ability of YhaO to transport D-serine we used an established CFT073 deletion mutant for known D-serine transporters, Δ*dsdXΔcycA* [23]. By transforming this strain with an inducible expression vector containing YhaO (*pyhaO-*ind) *from* EDL933 we could recover the wild-type feature and be certain that all transport of D-serine occurs through YhaO.

Cultures of *ΔdsdXΔcycA* + *pyhaO-*ind were grown in LB media overnight and used to inoculate MOPS media containing 3% glycerol at an OD_600_ of 0.1. The cultures were grown until an OD^600^ of 0.4 at which point 0.4 mM IPTG was added to initiate expression of *pyhaO-*ind. The cells were incubated for one hour at 37°C after induction and then harvested by centrifugation at 3500 g for 20 min. The cells were washed twice in PBS and resuspended in 500 μl PBS. The protein concentration of the sample was measured by QUBIT (Thermo-Scientific). Cells were split into 20 μl aliquots and kept on ice.

The transport of D-[^3^H]-serine was characterized by incubating cell suspensions with a range of concentrations of the radiolabelled ligand (0.1 μM, 1 μM and 10 μM) for 15 minutes. The cells were diluted in 5 ml MOPS-Tris buffer and then applied onto a membrane filter (0.2 μM). The liquid was moved through using a vacuum pump and the filters were washed in 5 ml MOPS-Tris buffer before the membranes were allowed to dry and placed in scintillation vials in 3 ml Biosafe counting cocktail (RPI Corp, Mount Prospect, IL, USA). The samples were measured in a scintillation counter. Time dependency of the D-serine uptake was measured by incubating samples with 1 μM D-[^3^H]-serine and diluting the samples into 5 ml MOPS-Tris at various time points (1 min to 20 min). Dependency of the transporter upon the membrane potential of the cell was confirmed by pretreatment of cell suspensions with 10 μM carbonyl cyanide m-chlorophenylhydrazone (CCCP) for three minutes. Competition assays were performed by pre-incubating the cell suspensions with a range of concentrations of L-serine or L-Threonine for three minutes before the incubation of 1 μM D-[^3^H]-serine for 3 minutes. Disintegrations-per-minute values determined by Scintillation counter. Data were normalized to the activity of D-[^3^H]-serine and to the overall protein concentration of the cell suspension using GraphPad Prism 5.0 (San Diego, CA, USA).

### Electrophoretic mobility shift assay (EMSA)

EMSA assays were performed using the DIG Gel Shift Kit, 2nd Generation and the DIG Wash and Block buffer set (Roche, Mannheim, Germany) according the manufacturers specifications with minor alterations. DNA probes were amplified by PCR using primers specific to the regions of interest. DNA fragments were labeled with ddUTP-11-DIG and diluted to a concentration of 0.4 ng/μl for use in binding reactions. Binding reactions were carried out for 30 minutes at 30°C using increasing concentrations of recombinant YhaJ (0 to 1 μM). Competition assays used a 100-fold excess of unlabeled specific or non-specific probe. A fragment of the *kan* gene was used as a non-specific control probe. Binding reactions were separated on 6% DNA retardation gels (Invitrogen) and transferred to positively charged nylon membrane (Roche) using the NOVEX system (Invitrogen). EMSA assays were then developed using AP conjugated anti-DIG antibody (Roche) according to the manufacturers specifications. EMSAs were repeated multiple times to confirm the results.

### Bioinformatic analysis of *dsdCXA* and *yhaOMKJ* carriage in *E*. *coli*

The nucleotide sequences for 159 *E*. *coli* core-genes were elaborated as described by previously [21,74]. At each iteration, the core gene set (initialised as the nucleotide sequence for genes present in strain MG1655) was aligned to the next *E*. *coli* genome sequence using blastn [75]. Genes aligning at > 70% identity and > 80% of the length of the coding sequence were retained in the core gene set for use in the next iteration. This analysis produced 159 genes, the nucleotide sequences of which were extracted from the *E*. *coli* genomes using blastn, aligned by Muscle [76] and concatenated. A maximum likelihood tree was constructed using PhyML under the GTR + g model of nucleotide substitution [77]. Dendrograms were visualized using the APE package [78] implemented in R [79].

The amino acid sequences for genes *yhaO*, *yhaM*, *yhaK* and *yhaJ* were collected from the EDL933 genome (NCBI accession number: NC_002655.2), and amino acid sequences for the genes *dsdX*, *dsdC* and *dsdA* were collected from the CFT073 genome (NCBI accession number: NC_004431.1). These sequences were used as the query sequence in tblastn searches against the *E*. *coli* genome sequences. In order to try to assemble hits which may be split over contigs, high scoring pairs (HSPs) aligning at greater than 70% amino acid similarity over greater than 30% of the coding sequence of the query protein were collected and assembled against the query sequence. The percent similarity (of the assembled hit to the query sequence) and coverage (of the assembled hit over the query sequence) was then calculated from these assembled hits and a total percent similarity between the hit and query sequence calculated by the formulae (percent similarity * coverage) / 100. The assembled hit sequences were saved for further pseudogene analysis. A gene was called as present within a genome if the assembled hit aligned to the query sequence at greater than 80% total similarity. The distribution of the genes across the *E*. *coli* core-genome dendrogram was visualized using the Diversitree package [80] implemented in R [79].

For the identification of pseudogenes, the recovered assembled hit sequences were investigated by both manual inspection and calculation of the length of the sequence until a stop codon was encountered. Obviously foreshortened or truncated proteins, those where a stop codon was encountered which shortened the protein by greater than 5% of the length of the query sequence, were assigned as pseudogenes.

## Supporting Information

S1 Fig Transcriptional context of the *yhaOMKJ* locus. (A) Primer design strategy for amplification of *yhaJ*, *yhaK*, *yhaM*, *yhaO* and *yhaM-yhaO* individual transcripts. (B) PCR amplification of each product from genomic DNA (gDNA) and complimentary DNA (cDNA) reverse transcribed from purified mRNA. Each product is color coded to correspond to the amplification strategy illustrated in panel A.
(TIF)

S2 Fig Growth and motility of *yhaO* and *yhaJ* deletion mutants generated in TUV93-0. (A) Growth of TUV93-0 and *ΔyhaO* in MEM-HEPES. (B) Motility of TUV93-0 and *ΔyhaO* on 0.25% Tryptone agar after 8 hours at 31°C. (C) Growth of TUV93-0 and *ΔyhaJ* in MEM-HEPES. (D) Motility of TUV93-0 and *ΔyhaJ* on 0.25% Tryptone agar after 8 hours at 31°C. Experiments were performed in triplicate.
(TIF)

S3 Fig RNA-seq analysis of NLE encoding elements in the EHEC genome. (A) RNA-seq read mapping to various NLE encoding cryptic prophage (CP) and O-islands (OI) in EHEC. The CP or OI being visualized is labeled above each coverage graph. NLEs are annotated below the relevant ORF and color-coded by the genomic element they are encoded upon. Transcript coverage for TUV93-0 is indicated by the grey peaks, whereas coverage peaks for the *yhaO* mutant are color coded by NLE encoding region. The graph height for each region comparison was scaled to TUV93-0 for direct comparison. Coverage graphs were generated from individual samples representative of three biological replicates. (B) Quantification of NLE differential expression represented as absolute fold change corresponding to the coverage graphs illustrated in panel A. Data was calculated using the EdgeR analysis tool implemented in CLC Genomics Workbench. *, ** and *** denote P ≤ 0.05, P ≤ 0.01 and P ≤ 0.001 respectively.
(TIF)

S4 Fig RNA-seq analysis of the LEE PAI in *ΔyhaO* and *ΔyhaJ*. Quantification of differential expression represented as absolute fold change from TUV93-0. Data was calculated from three biological replicates using the EdgeR analysis tool implemented in CLC Genomics Workbench. Red and orange bars represent *ΔyhaO* and *ΔyhaJ* respectively. Operonic structure of the LEE is indicated above the annotated ORFs.
(TIF)

S5 Fig Validation of RNA-seq data by qRT-PCR. The expression of *espD*, *tir*, *ler*, *nleA* and *nleG* in response to D-serine was investigated by analyzing relative mRNA transcript levels of each gene under LEE-inducing conditions. The wild type TUV93-0, *ΔyhaO* and *ΔyhaJ* backgrounds are indicated in grey, red and orange respectively. The red dashed line indicates relative baseline expression in TUV93-0, with genes expressed below this being down-regulated. * and ** denote P ≤ 0.05 and P ≤ 0.01 respectively calculated from 3 biological replicates.
(TIFF)

S6 Fig Assessment of the ability of YhaM from EHEC to metabolize D-serine. Comparison of TUV93-0 (EHEC), CFT073 (UPEC) and CFT073 *ΔdsdA* for the ability to grow on MOPS minimal agar plates containing D-serine as a sole carbon source. Complementation of *ΔdsdA* with either p*dsdA* from CFT073 but not p*yhaM* from EHEC restored the ability to grow on D-serine as a carbon source. Complementation of the wild type EHEC background with p*dsdA* from CFT073 also allowed growth on D-serine as a carbon source.
(TIF)

S7 Fig EMSA analysis of distinct NLE promoter regions incubated with purified YhaJ. Approximately 400 bp DNA fragments corresponding to upstream regions of *nleA*, *nleB*, *nleG1* and *nleG6* were DIG-labeled and incubated with 1 μM YhaJ for EMSA analysis (+). As a control, DNA fragments alone were ran beside binding reactions (-). No visible band shift was observed when YhaJ was incubated with any of the DNA fragments tested suggesting that YhaJ does not directly interact with NLE upstream regulatory regions. The black arrow indicates free DNA.
(TIF)

S1 Table Pseudogene accumulation in *yhaOMKJ* of *E*. *coli* and *Shigella*. (DOCX)

S2 Table Differentially expressed genes identified between *ΔyhaO* and *ΔyhaJ* and TUV93-0 by RNA-seq (MEM-HEPES; OD ~0.8). (DOCX)

S3 Table Strains used in this study. (DOCX)

S4 Table Plasmids used in this study. (DOCX)

S5 Table Primers used in this study. (DOCX)
