# Supplementary figures and images for: A Highly Conserved Bacterial D-Serine Uptake System Links Host Metabolism and Virulence
Source: PLoS Pathog. 2016 Jan 4;12(1):e1005359. doi: 10.1371/journal.ppat.1005359 (PMC4699771; doi:10.1371/journal.ppat.1005359)

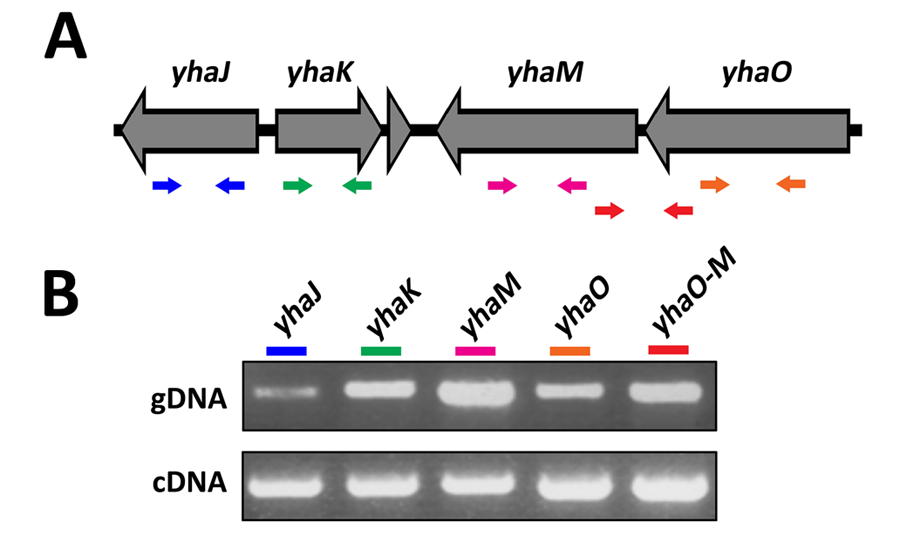

Supplement: S1 Fig — (A) Primer design strategy for amplification of yhaJ, yhaK, yhaM, yhaO and yhaM-yhaO individual transcripts. (B) PCR amplification of each product from genomic DNA (gDNA) and complimentary DNA (cDNA) reverse transcribed from purified mRNA. Each product is color coded to correspond to the amplification strategy illustrated in panel A. (TIF) [file ppat.1005359.s001.tif]

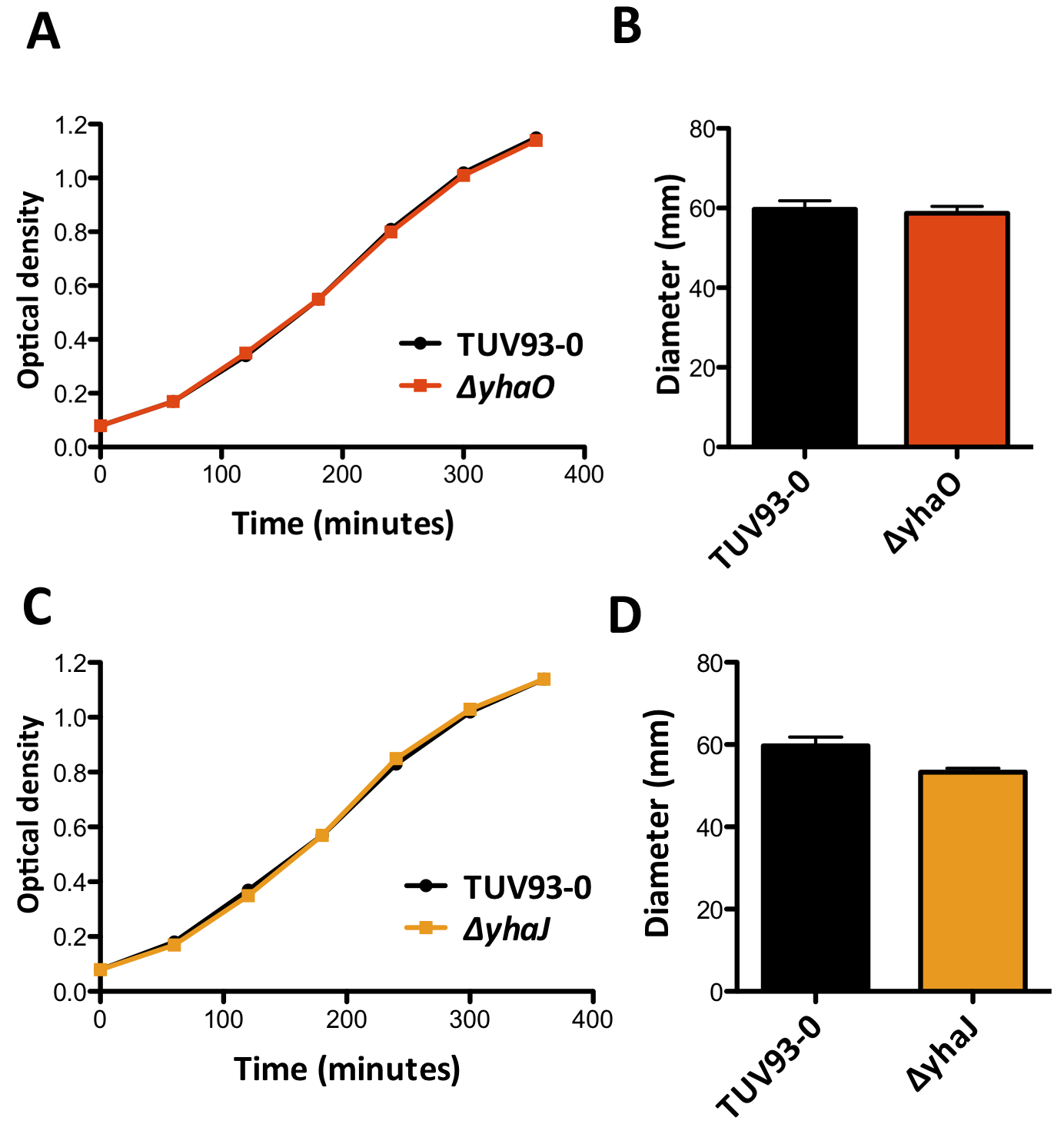

Supplement: S2 Fig — (A) Growth of TUV93-0 and ΔyhaO in MEM-HEPES. (B) Motility of TUV93-0 and ΔyhaO on 0.25% Tryptone agar after 8 hours at 31°C. (C) Growth of TUV93-0 and ΔyhaJ in MEM-HEPES. (D) Motility of TUV93-0 and ΔyhaJ on 0.25% Tryptone agar after 8 hours at 31°C. Experiments were performed in triplicate. (TIF) [file ppat.1005359.s002.tif]

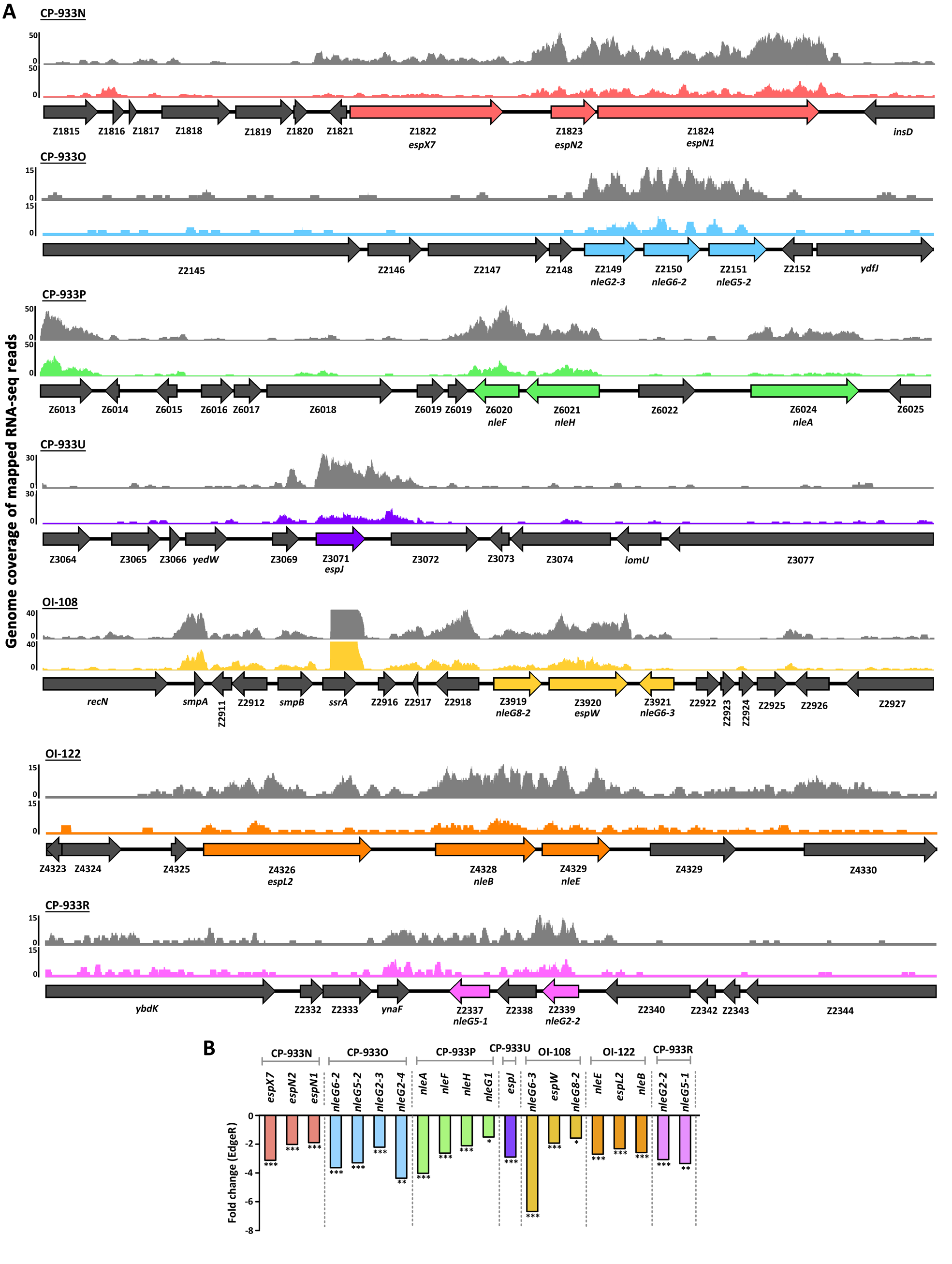

Supplement: S3 Fig — (A) RNA-seq read mapping to various NLE encoding cryptic prophage (CP) and O-islands (OI) in EHEC. The CP or OI being visualized is labeled above each coverage graph. NLEs are annotated below the relevant ORF and color-coded by the genomic element they are encoded upon. Transcript coverage for TUV93-0 is indicated by the grey peaks, whereas coverage peaks for the yhaO mutant are color coded by NLE encoding region. The graph height for each region comparison was scaled to TUV93-0 for direct comparison. Coverage graphs were generated from individual samples representative of three biological replicates. (B) Quantification of NLE differential expression represented as absolute fold change corresponding to the coverage graphs illustrated in panel A. Data was calculated using the EdgeR analysis tool implemented in CLC Genomics Workbench. *, ** and *** denote P ≤ 0.05, P ≤ 0.01 and P ≤ 0.001 respectively. (TIF) [file ppat.1005359.s003.tif]

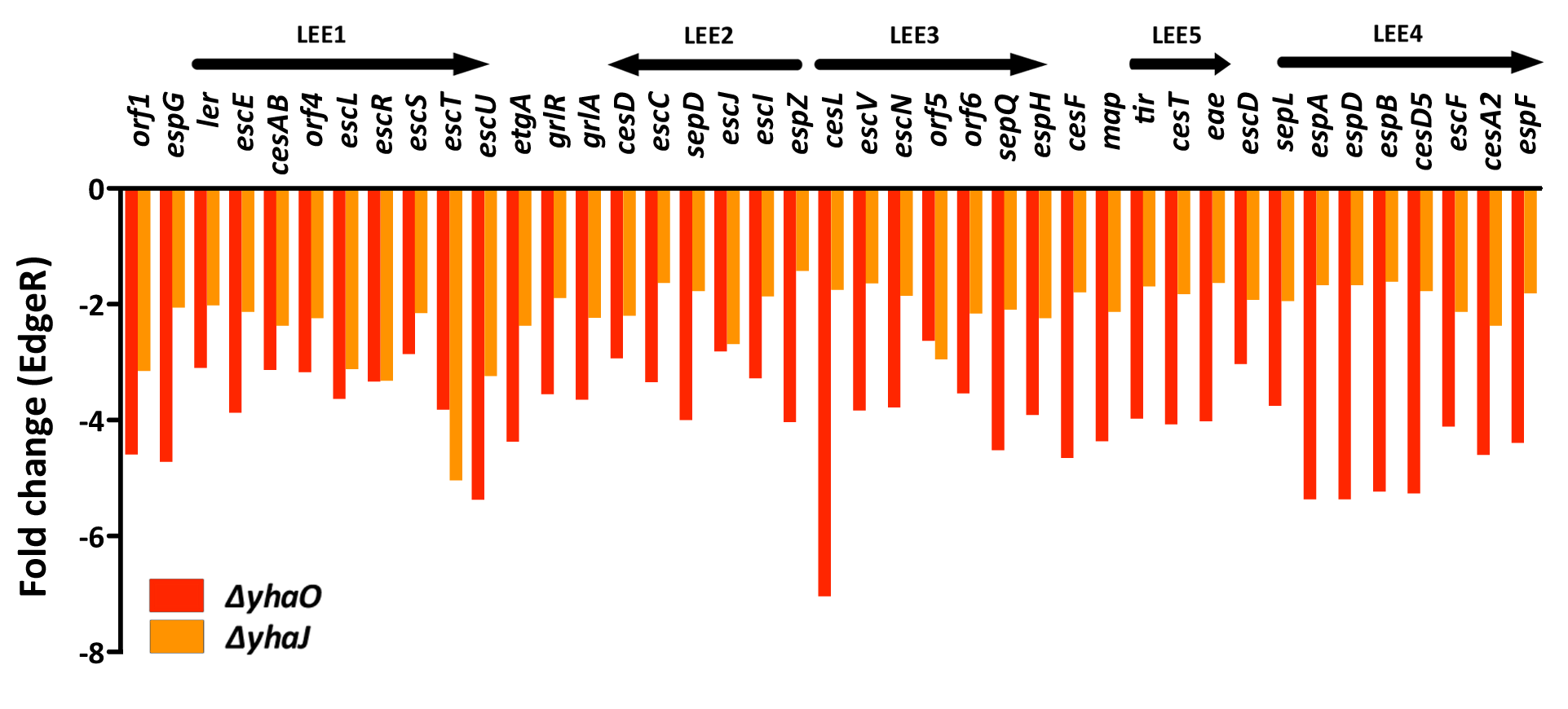

Supplement: S4 Fig — Quantification of differential expression represented as absolute fold change from TUV93-0. Data was calculated from three biological replicates using the EdgeR analysis tool implemented in CLC Genomics Workbench. Red and orange bars represent ΔyhaO and ΔyhaJ respectively. Operonic structure of the LEE is indicated above the annotated ORFs. (TIF) [file ppat.1005359.s004.tif]

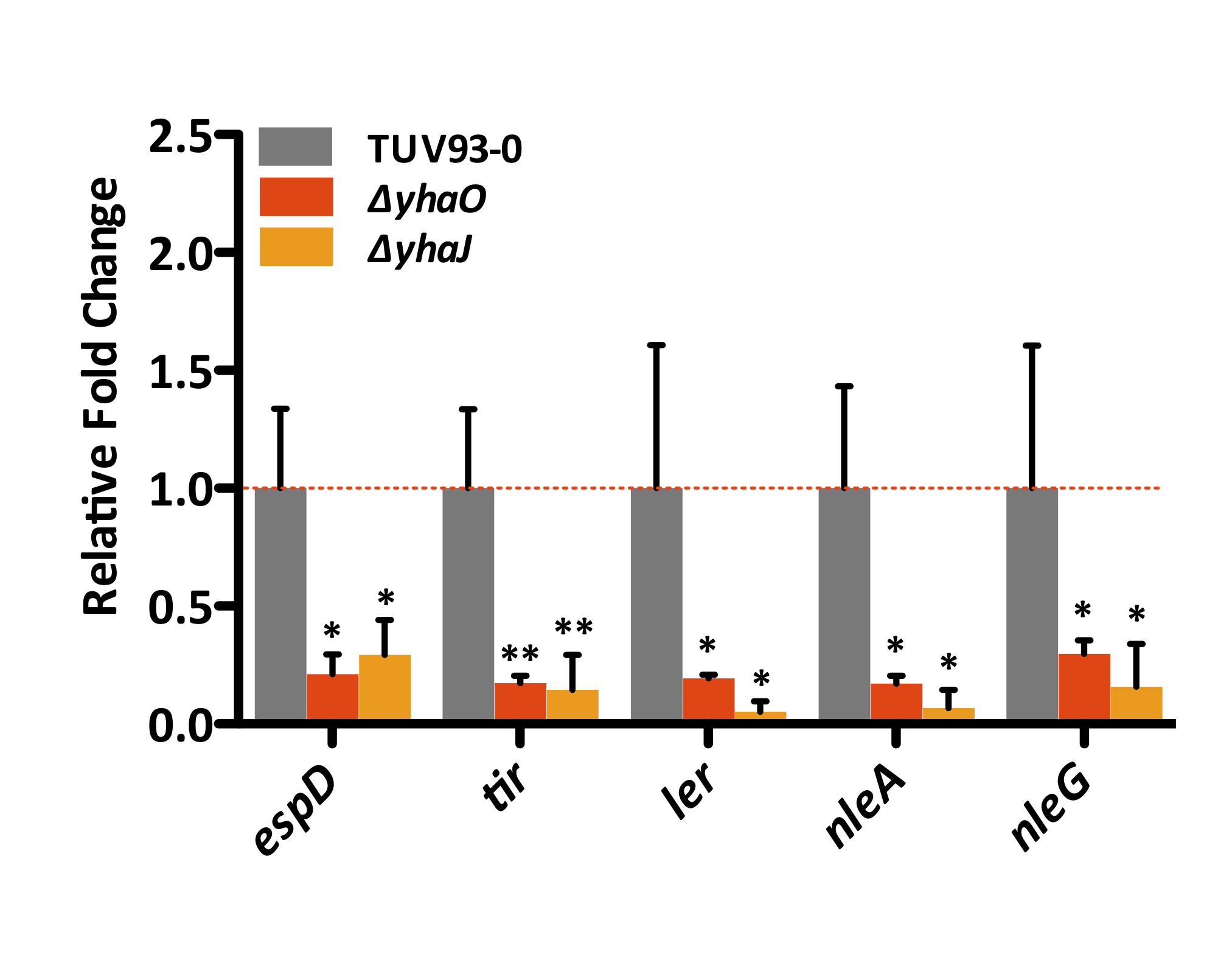

Supplement: S5 Fig — The expression of espD, tir, ler, nleA and nleG in response to D-serine was investigated by analyzing relative mRNA transcript levels of each gene under LEE-inducing conditions. The wild type TUV93-0, ΔyhaO and ΔyhaJ backgrounds are indicated in grey, red and orange respectively. The red dashed line indicates relative baseline expression in TUV93-0, with genes expressed below this being down-regulated. * and ** denote P ≤ 0.05 and P ≤ 0.01 respectively calculated from 3 biological replicates. (TIFF) [file ppat.1005359.s005.tiff]

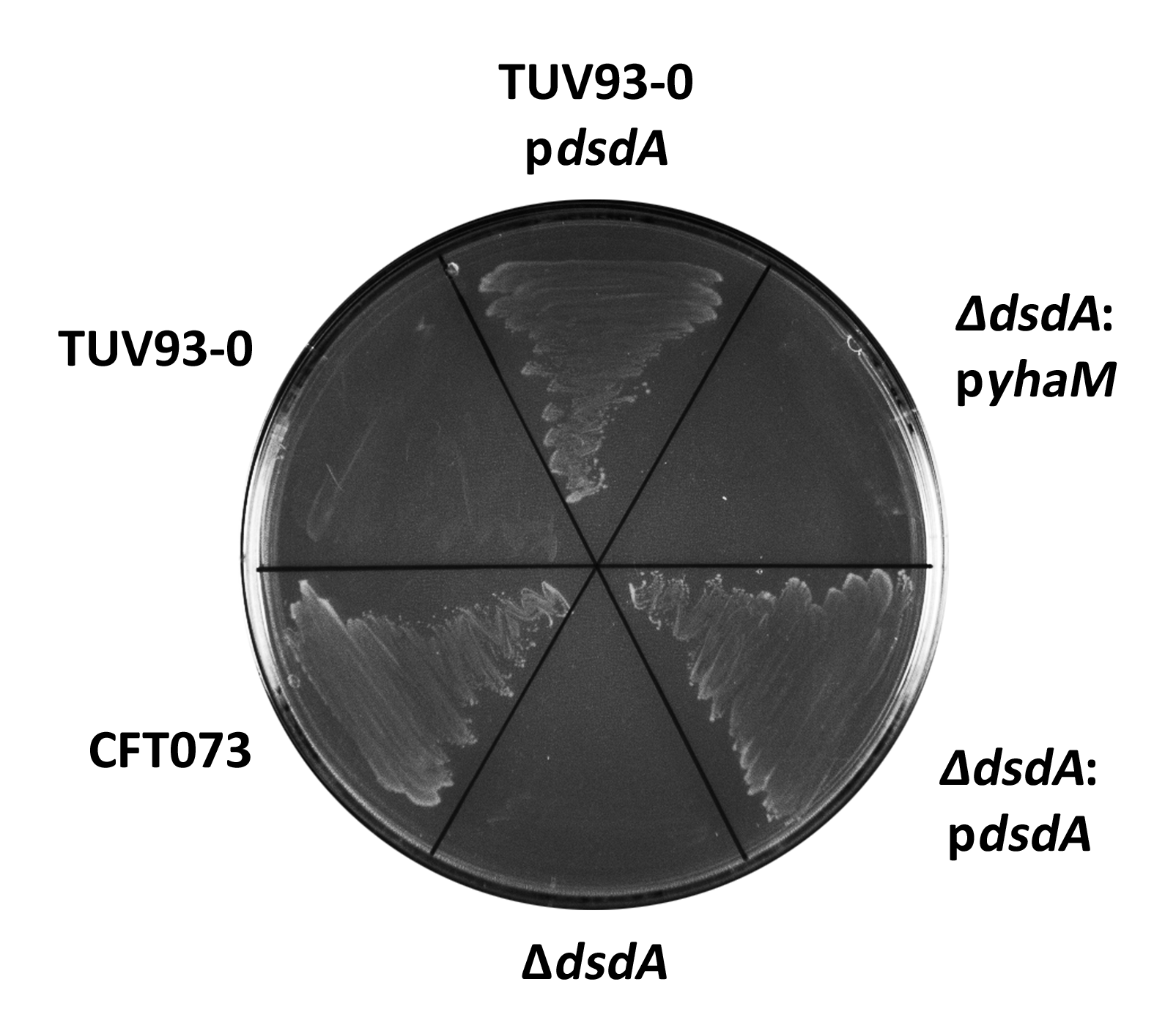

Supplement: S6 Fig — Comparison of TUV93-0 (EHEC), CFT073 (UPEC) and CFT073 ΔdsdA for the ability to grow on MOPS minimal agar plates containing D-serine as a sole carbon source. Complementation of ΔdsdA with either pdsdA from CFT073 but not pyhaM from EHEC restored the ability to grow on D-serine as a carbon source. Complementation of the wild type EHEC background with pdsdA from CFT073 also allowed growth on D-serine as a carbon source. (TIF) [file ppat.1005359.s006.tif]

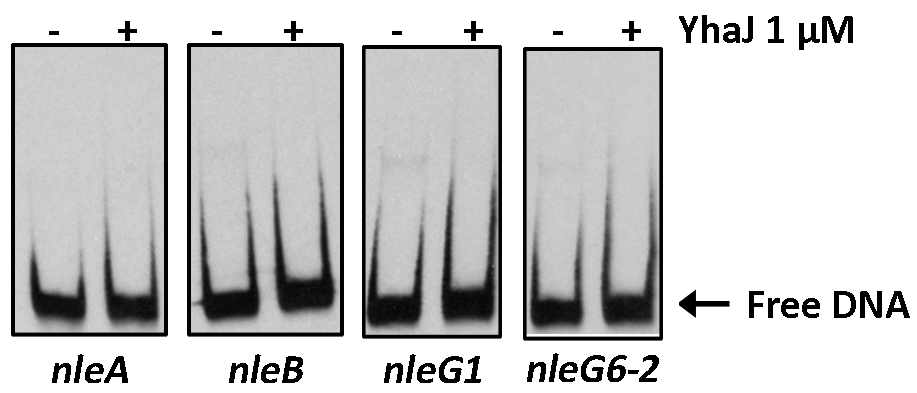

Supplement: S7 Fig — Approximately 400 bp DNA fragments corresponding to upstream regions of nleA, nleB, nleG1 and nleG6 were DIG-labeled and incubated with 1 μM YhaJ for EMSA analysis (+). As a control, DNA fragments alone were ran beside binding reactions (-). No visible band shift was observed when YhaJ was incubated with any of the DNA fragments tested suggesting that YhaJ does not directly interact with NLE upstream regulatory regions. The black arrow indicates free DNA. (TIF) [file ppat.1005359.s007.tif]
