# Supplementary material for: A Highly Conserved Bacterial D-Serine Uptake System Links Host Metabolism and Virulence
Source: PLoS Pathog. 2016 Jan 4;12(1):e1005359. doi: 10.1371/journal.ppat.1005359 (PMC4699771; doi:10.1371/journal.ppat.1005359)
Supplement: S1 Table — (DOCX) [file ppat.1005359.s008.docx]

| **Table S1. Pseudogene accumulation in *yhaOMKJ* of *E. coli* and *Shigella*** | | | | |  |
| --- | --- | --- | --- | --- | --- |
| **Gene** | **Number of pseudogenes in all *E. coli*** | **% of all *E. coli* with pseudogene (1581 genomes)** | **Number of pseudogenes in *Shigella*** | **% of *Shigella* with pseudogene (45 genomes)** | **% of all pseudogenes are because of *Shigella*** |
| *yhaO* | 52 | 3.29 | 8 | 17.78 | 15.38 |
| *yhaM* | 7 | 0.44 | 1 | 2.22 | 14.29 |
| *yhaK* | 0 | 0 | 0 | 0 | 0 |
| *yhaJ* | 29 | 1.83 | 25 | 55.56 | 86.21 |
