# Supplementary material for: A Highly Conserved Bacterial D-Serine Uptake System Links Host Metabolism and Virulence
Source: PLoS Pathog. 2016 Jan 4;12(1):e1005359. doi: 10.1371/journal.ppat.1005359 (PMC4699771; doi:10.1371/journal.ppat.1005359)
Supplement: S2 Table — (DOCX) [file ppat.1005359.s009.docx]

| **Table S2. Differentially expressed genes identified in *ΔyhaO* and *ΔyhaJ* by RNA-seq (MEM-HEPES; OD ~0.8)** | | | | | |
| --- | --- | --- | --- | --- | --- |
|  | **Gene ID (EDL933)** | **Fold change** | **FDR p-val** | **Mean expression TUV93-0** | **Mean expression mutant** |
| ***ΔyhaO*** | *Z4325* | -35.2 | 0.03 | 4 | 0 |
|  | *Z5121* | -7.04 | 5.52E-07 | 19.67 | 3 |
|  | *Z3921* | -6.68 | 6.88E-09 | 21 | 3.33 |
|  | *Z1821* | -5.97 | 1.49E-03 | 9.67 | 1.67 |
|  | *escU* | -5.37 | 5.27E-12 | 38.33 | 7.67 |
|  | *espA* | -5.36 | 0 | 3,605.00 | 743.33 |
|  | *cesD2* | -5.26 | 7.29E-36 | 1,151.67 | 243 |
|  | *espB* | -5.23 | 0 | 5,494.00 | 1,164.33 |
|  | *espD* | -5.01 | 0 | 5,349.33 | 1,187.67 |
|  | *Z5142* | -4.72 | 1.17E-18 | 187.67 | 44.67 |
|  | *Z5114* | -4.65 | 1.75E-15 | 89.67 | 21.33 |
|  | *Z5102* | -4.6 | 6.44E-19 | 155 | 37.33 |
|  | *Z5143* | -4.59 | 5.52E-07 | 32 | 7.67 |
|  | *sepQ* | -4.52 | 1.02E-24 | 225.33 | 55.67 |
|  | *Z6019* | -4.5 | 2.70E-03 | 10 | 2.33 |
|  | *Z0957* | -4.48 | 1.11E-07 | 56.67 | 14 |
|  | *espF* | -4.39 | 4.06E-28 | 395.33 | 99.67 |
|  | *Z5131* | -4.37 | 3.37E-06 | 19 | 4.67 |
|  | *Z2075* | -4.36 | 4.51E-03 | 9.67 | 2.33 |
|  | *Z5113* | -4.36 | 2.54E-27 | 207.67 | 53 |
|  | *mtr* | -4.16 | 3.59E-08 | 33 | 8.67 |
|  | *escF* | -4.11 | 1.11E-17 | 164 | 44 |
|  | *Z5111* | -4.07 | 1.80E-37 | 760.67 | 205 |
|  | *sepZ* | -4.03 | 2.55E-21 | 427.33 | 118.67 |
|  | *eae* | -4.02 | 0 | 5,977.33 | 1,623.33 |
|  | *Z6024* | -4.02 | 1.80E-13 | 109.67 | 30 |
|  | *Z5125* | -4 | 3.66E-27 | 111 | 30.33 |
|  | *tir* | -3.97 | 0 | 2,347.67 | 654 |
|  | *Z5115* | -3.91 | 1.36E-22 | 215.33 | 60.67 |
|  | *Z5139* | -3.87 | 6.07E-16 | 59 | 16.67 |
|  | *escV* | -3.83 | 1.21E-15 | 237.67 | 69 |
|  | *escT* | -3.82 | 9.51E-13 | 45.67 | 13 |
|  | *escN* | -3.78 | 3.88E-29 | 261.33 | 75.67 |
|  | *sepL* | -3.75 | 1.19E-30 | 558.33 | 165.33 |
|  | *Z5128* | -3.64 | 9.42E-25 | 117.67 | 35.67 |
|  | *Z5136* | -3.63 | 7.21E-23 | 98.33 | 29.67 |
|  | *Z2150* | -3.63 | 2.72E-05 | 37.33 | 11.33 |
|  | *Z0955* | -3.59 | 1.78E-21 | 493 | 150 |
|  | *Z5129* | -3.55 | 1.40E-14 | 203.67 | 63.33 |
|  | *Z5117* | -3.54 | 2.33E-04 | 24.33 | 7.67 |
|  | *escC* | -3.34 | 4.17E-20 | 330 | 108.67 |
|  | *Z2337* | -3.34 | 4.04E-03 | 19 | 6.33 |
|  | *escR* | -3.33 | 4.72E-10 | 60 | 20 |
|  | *Z2151* | -3.29 | 1.47E-04 | 21.33 | 7 |
|  | *Z5123* | -3.28 | 5.94E-12 | 159.33 | 53 |
|  | *Z5137* | -3.17 | 1.14E-15 | 168.33 | 59 |
|  | *Z5138* | -3.13 | 1.60E-12 | 71 | 24.67 |
|  | *Z1822* | -3.12 | 1.83E-11 | 79.33 | 28.33 |
|  | *Z5140* | -3.1 | 1.78E-14 | 144.67 | 51.67 |
|  | *Z2339* | -3.07 | 6.08E-04 | 18 | 6.33 |
|  | *escD* | -3.03 | 5.98E-14 | 148.33 | 54.67 |
|  | *cesD* | -2.93 | 1.78E-14 | 97.33 | 36.67 |
|  | *Z3071* | -2.89 | 1.11E-17 | 110.67 | 41.67 |
|  | *escS* | -2.86 | 1.41E-04 | 23.67 | 9 |
|  | *escJ* | -2.81 | 1.60E-10 | 119 | 47 |
|  | *Z4329* | -2.69 | 3.36E-05 | 34.67 | 14 |
|  | *Z5118* | -2.63 | 5.58E-04 | 44.67 | 19 |
|  | *Z6020* | -2.62 | 4.72E-10 | 130.33 | 55 |
|  | *Z0956* | -2.6 | 0.02 | 16 | 6.67 |
|  | *Z4328* | -2.58 | 4.59E-04 | 67.33 | 29 |
|  | *leuO* | -2.54 | 0.03 | 13.33 | 5.67 |
|  | *Z4332* | -2.5 | 1.26E-05 | 36.67 | 16 |
|  | *zraP* | -2.31 | 0.03 | 17 | 8 |
|  | *Z4326* | -2.31 | 6.14E-05 | 57.67 | 27.67 |
|  | *Z2149* | -2.2 | 9.34E-04 | 43.33 | 21.33 |
|  | *Z1793* | -2.13 | 3.35E-03 | 30.67 | 15.67 |
|  | *Z6021* | -2.1 | 4.10E-09 | 105 | 54.67 |
|  | *pgaA* | -2.04 | 0.02 | 31.33 | 16.67 |
|  | *Z1824* | -2.01 | 9.28E-10 | 378.33 | 206.67 |
|  | *rcsA* | -1.92 | 3.69E-05 | 75 | 43 |
|  | *Z3920* | -1.92 | 5.06E-07 | 186.33 | 107.33 |
|  | *Z1823* | -1.89 | 8.90E-05 | 59.33 | 34.33 |
|  | *wcaG* | -1.86 | 0.01 | 32.67 | 19.33 |
|  | *hypD* | -1.83 | 0.02 | 31.33 | 18.67 |
|  | *gmd* | -1.77 | 5.36E-03 | 55 | 34 |
|  | *yfiA* | -1.71 | 3.09E-05 | 308.67 | 198.67 |
|  | *Z3931* | -1.6 | 0.02 | 50.67 | 34.67 |
|  | *yhcK* | -1.59 | 0.04 | 49 | 33.67 |
|  | *Z3919* | -1.57 | 0.04 | 45.33 | 31.67 |
|  | *trpB* | -1.54 | 0.02 | 75.67 | 54 |
|  | *proP* | -1.51 | 4.08E-03 | 383.67 | 278.33 |
|  | *Z6010* | -1.5 | 0.04 | 107.33 | 78 |
|  | *Z4849* | -1.5 | 0.02 | 75.67 | 55.33 |
|  | *Z1341* | -1.5 | 0.02 | 165.67 | 121.33 |
|  | *ycdB* | 1.51 | 4.44E-03 | 210 | 342.33 |
|  | *entC* | 1.57 | 5.16E-04 | 267.67 | 458.33 |
|  | *speD* | 1.57 | 0.01 | 43.67 | 75.33 |
|  | *nrdH* | 1.58 | 5.92E-03 | 55.33 | 96 |
|  | *rmf* | 1.6 | 2.69E-04 | 557 | 986.33 |
|  | *Z4386* | 1.61 | 2.09E-03 | 51.33 | 90 |
|  | *pncB* | 1.61 | 1.17E-03 | 118 | 208.33 |
|  | *metK* | 1.61 | 8.10E-04 | 130 | 230.67 |
|  | *livJ* | 1.62 | 5.44E-03 | 204.67 | 357.67 |
|  | *csgD* | 1.71 | 0.04 | 25 | 47 |
|  | *Z3561* | 1.74 | 0.05 | 21.67 | 41.33 |
|  | *htrL* | 1.74 | 0.04 | 20 | 38.33 |
|  | *moaE* | 1.84 | 8.18E-04 | 26.33 | 53.33 |
|  | *moaC* | 1.87 | 0.02 | 29.33 | 58.67 |
|  | *metN* | 1.88 | 4.78E-03 | 22.33 | 46 |
|  | *nadB* | 2.2 | 4.07E-04 | 16.67 | 40.33 |
|  | *metE* | 2.57 | 1.92E-17 | 254 | 703.33 |
|  | *Z3023* | 2.7 | 0.01 | 6 | 18 |
|  | *Z2082* | 2.76 | 0.03 | 4.67 | 14.33 |
|  | *metF* | 3.95 | 4.97E-06 | 6 | 26 |
|  | *Z4462* | 5.61 | 6.42E-104 | 153 | 933.67 |
|  | *Z2351* | 29.04 | 0.04 | 0 | 3.67 |
|  |  |  |  |  |  |
| ***ΔyhaJ*** | *thrL* | -5.5 | 0.04 | 9 | 1.33 |
|  | *escT* | -5.04 | 2.25E-07 | 51 | 8.67 |
|  | *Z2873* | -4.73 | 0.03 | 7.67 | 1.33 |
|  | *Z0397* | -4.36 | 2.05E-03 | 13.67 | 2.67 |
|  | *Z6025* | -4.14 | 1.77E-04 | 17.67 | 3.67 |
|  | *Z0405* | -3.42 | 0.03 | 12 | 3 |
|  | *Z4314* | -3.39 | 8.08E-03 | 20.67 | 5.33 |
|  | *escR* | -3.32 | 1.72E-07 | 66.67 | 17.33 |
|  | *escU* | -3.24 | 1.49E-05 | 47.67 | 12.67 |
|  | *Z5143* | -3.15 | 1.74E-04 | 36.67 | 10 |
|  | *Z5136* | -3.12 | 1.59E-09 | 158.33 | 44 |
|  | *Z5118* | -2.95 | 7.68E-07 | 59 | 17.33 |
|  | *Z5488* | -2.86 | 8.29E-03 | 19.67 | 6 |
|  | *ylaD* | -2.75 | 1.26E-03 | 26.33 | 8.33 |
|  | *Z3071* | -2.72 | 9.26E-08 | 105.67 | 33.67 |
|  | *escJ* | -2.68 | 6.21E-06 | 229.33 | 73 |
|  | *acpD* | -2.63 | 1.84E-05 | 50.33 | 16.67 |
|  | *fxsA* | -2.61 | 1.93E-03 | 28 | 9.33 |
|  | *Z5187* | -2.53 | 0.01 | 26.33 | 9 |
|  | *Z1823* | -2.47 | 0.04 | 18 | 6.33 |
|  | *Z3768* | -2.4 | 2.41E-03 | 64 | 23.33 |
|  | *Z5131* | -2.37 | 0.01 | 29 | 10.67 |
|  | *Z5138* | -2.37 | 1.12E-05 | 116 | 42.33 |
|  | *Z5102* | -2.37 | 1.77E-04 | 297.33 | 107.67 |
|  | *Z2974* | -2.36 | 0.03 | 21.67 | 8 |
|  | *yheA* | -2.25 | 1.25E-04 | 76.67 | 29.67 |
|  | *sdhC* | -2.25 | 8.94E-03 | 73.33 | 28 |
|  | *Z5137* | -2.24 | 2.68E-07 | 226.67 | 87.67 |
|  | *Z5115* | -2.24 | 8.40E-06 | 301.33 | 115.67 |
|  | *Z5128* | -2.23 | 2.01E-04 | 149.33 | 57.33 |
|  | *evgA* | -2.22 | 0.04 | 23.67 | 9.33 |
|  | *cesD* | -2.2 | 2.87E-07 | 206.33 | 81.33 |
|  | *Z5117* | -2.16 | 0.01 | 33 | 13.33 |
|  | *Z0955* | -2.15 | 2.39E-07 | 861 | 349.67 |
|  | *Z5113* | -2.13 | 8.46E-07 | 422.67 | 171 |
|  | *escF* | -2.13 | 1.17E-04 | 239.33 | 96.33 |
|  | *Z5139* | -2.13 | 3.57E-03 | 74 | 30 |
|  | *Z3931* | -2.12 | 9.27E-04 | 172.67 | 70 |
|  | *sepQ* | -2.09 | 2.36E-05 | 221.67 | 92.33 |
|  | *Z1074* | -2.06 | 0.01 | 33 | 14 |
|  | *Z5142* | -2.06 | 3.42E-04 | 223.33 | 94.33 |
|  | *Z1693* | -2.04 | 0.05 | 25.67 | 11 |
|  | *Z0985* | -2.03 | 0.02 | 33.33 | 14.33 |
|  | *Z5140* | -2.02 | 1.90E-05 | 298.33 | 127.33 |
|  | *nuoJ* | -1.97 | 0.02 | 39.67 | 17.67 |
|  | *Z0957* | -1.95 | 6.87E-03 | 134.33 | 60 |
|  | *Z1625* | -1.95 | 1.24E-03 | 57 | 25.67 |
|  | *sepL* | -1.94 | 1.22E-04 | 1,038.67 | 460.67 |
|  | *pitB* | -1.93 | 0.04 | 31.67 | 14.33 |
|  | *escD* | -1.92 | 2.37E-04 | 262.33 | 119 |
|  | *Z6024* | -1.9 | 2.67E-04 | 146.33 | 66.67 |
|  | *Z5129* | -1.89 | 1.74E-04 | 388 | 176.67 |
|  | *Z5123* | -1.86 | 3.68E-05 | 341.67 | 159.67 |
|  | *escN* | -1.85 | 1.20E-05 | 377 | 178.33 |
|  | *bax* | -1.83 | 4.71E-05 | 189.33 | 91 |
|  | *Z5111* | -1.82 | 4.49E-04 | 914.67 | 436.67 |
|  | *espF* | -1.81 | 1.53E-03 | 850 | 410 |
|  | *stpA* | -1.8 | 0.04 | 44.33 | 21.67 |
|  | *Z5114* | -1.79 | 3.43E-05 | 155 | 76 |
|  | *Z5125* | -1.77 | 3.13E-05 | 245.67 | 121.67 |
|  | *Z5104* | -1.77 | 1.26E-03 | 2,155.33 | 1,058.67 |
|  | *Z1824* | -1.76 | 1.28E-03 | 111.67 | 55.67 |
|  | *clpS* | -1.73 | 0.05 | 51.33 | 26 |
|  | *secG* | -1.72 | 1.02E-03 | 350.33 | 177 |
|  | *yciS* | -1.7 | 0.02 | 52.67 | 27.33 |
|  | *tir* | -1.69 | 1.71E-03 | 4,678.00 | 2,419.33 |
|  | *espA* | -1.67 | 7.34E-03 | 8,699.00 | 4,508.67 |
|  | *espD* | -1.67 | 7.02E-03 | 15,679.33 | 8,125.00 |
|  | *escV* | -1.64 | 6.08E-05 | 360.67 | 192.67 |
|  | *eae* | -1.63 | 8.84E-03 | 7,683.00 | 4,133.33 |
|  | *escC* | -1.63 | 1.35E-03 | 592.33 | 316 |
|  | *yejG* | -1.61 | 0.03 | 81 | 44 |
|  | *espB* | -1.61 | 0.01 | 13,892.67 | 7,500.67 |
|  | *infA* | -1.61 | 0.04 | 140.67 | 77 |
|  | *Z4328* | -1.55 | 6.87E-03 | 121.33 | 68.67 |
|  | *uvrY* | -1.54 | 9.46E-03 | 135 | 77 |
|  | *yihE* | -1.51 | 0.02 | 87.33 | 51 |
|  | *ybaY* | 1.57 | 2.11E-03 | 112.33 | 155.33 |
|  | *Z4454* | 1.57 | 0.05 | 47.33 | 65.67 |
|  | *gcd* | 1.57 | 8.30E-03 | 102.67 | 143.33 |
|  | *yqjC* | 1.58 | 0.02 | 40.67 | 56.67 |
|  | *poxB* | 1.67 | 4.72E-04 | 86 | 127.67 |
|  | *Z2754* | 1.67 | 0.05 | 37 | 54 |
|  | *katE* | 1.72 | 1.66E-03 | 60.33 | 92 |
|  | *lysA* | 1.72 | 4.62E-03 | 43.67 | 66.67 |
|  | *dps* | 1.74 | 2.87E-07 | 941 | 1,462.67 |
|  | *yqjD* | 1.75 | 1.73E-03 | 55.33 | 86.33 |
|  | *ydaJ* | 1.82 | 1.28E-03 | 103 | 167 |
|  | *ybaS* | 1.83 | 8.30E-03 | 28.33 | 45.67 |
|  | *msyB* | 1.84 | 0.02 | 22.33 | 36.33 |
|  | *slp* | 1.89 | 2.46E-07 | 377 | 637 |
|  | *ecnB* | 1.91 | 3.57E-03 | 32 | 54.33 |
|  | *ompC* | 2.04 | 1.18E-06 | 3,563.33 | 6,550.33 |
|  | *yhiV* | 2.32 | 8.68E-11 | 91 | 187.67 |
|  | *yhiU* | 2.46 | 2.36E-05 | 32.67 | 72 |
|  | *hdeA* | 2.5 | 1.85E-08 | 472 | 1,069.33 |
|  | *csgA* | 2.53 | 4.38E-10 | 186 | 419.67 |
|  | *hdeD* | 2.63 | 5.55E-08 | 75 | 177 |
|  | *csgB* | 2.69 | 1.23E-05 | 53.67 | 130 |
|  | *metE* | 2.71 | 2.94E-06 | 538 | 1,338.33 |
|  | *yhiM* | 2.76 | 4.41E-05 | 31.33 | 76.33 |
|  | *gadA* | 2.78 | 7.43E-10 | 236.33 | 595.33 |
|  | *gadB* | 3.08 | 5.25E-18 | 453 | 1,250.67 |
|  | *xasA* | 3.12 | 3.69E-10 | 349 | 991.33 |
|  | *frdC* | 3.9 | 0.04 | 2.33 | 8.33 |
|  | *yqjF* | 4.71 | 0.03 | 1.67 | 7.33 |
