# Supplementary material for: A Highly Conserved Bacterial D-Serine Uptake System Links Host Metabolism and Virulence
Source: PLoS Pathog. 2016 Jan 4;12(1):e1005359. doi: 10.1371/journal.ppat.1005359 (PMC4699771; doi:10.1371/journal.ppat.1005359)
Supplement: S3 Table — (DOCX) [file ppat.1005359.s010.docx]

| **Table S3. Strains used in this study** |  |  |
| --- | --- | --- |
| **Name** | **Description** | **Source** |
| EHEC TUV93-0 | Wild type *E. coli* O157:H7 derived from EDL933 (shiga toxin negative) | Roe lab inventory |
| *ΔyhaO* | TUV93-0 *yhaO deletion* mutant | Roe lab inventory |
| *ΔyhaM* | TUV93-0 *yhaM deletion* mutant | Roe lab inventory |
| *ΔyhaK* | TUV93-0 *yhaK deletion* mutant | Roe lab inventory |
| *ΔyhaJ* | TUV93-0 *yhaJ deletion* mutant | Roe lab inventory |
| UPEC | Wild type *E. coli* CFT073 | Anfora & Welch 2006 |
| UPEC Δ*dsdA* | CFT073 *dsdA* mutant | Anfora & Welch 2006 |
| UPEC Δ*dsdX*Δ*cycA* | CFT073 *dsdX cycA* double mutant | Anfora & Welch 2006 |
