# Supplementary material for: A Highly Conserved Bacterial D-Serine Uptake System Links Host Metabolism and Virulence
Source: PLoS Pathog. 2016 Jan 4;12(1):e1005359. doi: 10.1371/journal.ppat.1005359 (PMC4699771; doi:10.1371/journal.ppat.1005359)
Supplement: S4 Table — (DOCX) [file ppat.1005359.s011.docx]

| **Table S4. Plasmids used in this study** |
| --- |

| **Name** | **Description** | **Source** |
| --- | --- | --- |
| p*yhaO* | pWSK29-*yhaO* complementation plasmid (Ampicillin) | This study |
| p*yhaM* | pWSK29-*yhaM* complementation plasmid (Ampicillin) | This study |
| p*yhaJ* | pWSK29-*yhaJ* complementation plasmid (Ampicillin) | This study |
| p*yhaO*:GFP | Promoter region of *yhaO* from EDL933 fused in-frame to eGFP (Kanamycin) | Genscript |
| p*dsdC*:GFP | Promoter region of *dsdC* from K-12 fused in-frame to GFP in pUA66 (Kanamycin) | Zaslaver *et al.* 2006 |
| p*dsdX*:GFP | Promoter region of *dsdX* from K-12 fused in-frame to GFP in pUA66 (Kanamycin) | Zaslaver *et al.* 2006 |
| p*yhaJ-*His | *yhaJ* from EDL933 cloned into pET-28b (Kanamycin) | This study |
| p*dsdA* | pACYC-*dsdA* complementation construct | Anfora & Welch 2006 |
| p*dsdX* | pACYC-*dsdX* complementation construct | Anfora & Welch 2006 |
| pRFP | Constitutively expressed RFP plasmid | Roe lab inventory |
| LEE10-568 | 568 base pairs upstream of the LEE1 coding region fused to *lacZ* | Islam et al. 2011 |
| LEE10-275 | 275 base pairs upstream of the LEE1 coding region fused to *lacZ* | Islam et al. 2011 |
| LEE10-155 | 155 base pairs upstream of the LEE1 coding region fused to *lacZ* | Islam et al. 2011 |
| LEE10-115 | 115 base pairs upstream of the LEE1 coding region fused to *lacZ* | Islam et al. 2011 |
| LEE20-568 | Base pairs -158 to -568 upstream of the LEE1 coding region fused to *lacZ* | Islam et al. 2011 |
| LEE20-275 | Base pairs -158 to -275 upstream of the LEE1 coding region fused to *lacZ* | Islam et al. 2011 |
