# Supplementary material for: A Highly Conserved Bacterial D-Serine Uptake System Links Host Metabolism and Virulence
Source: PLoS Pathog. 2016 Jan 4;12(1):e1005359. doi: 10.1371/journal.ppat.1005359 (PMC4699771; doi:10.1371/journal.ppat.1005359)
Supplement: S5 Table — (DOCX) [file ppat.1005359.s012.docx]

| **Table S5. Primers used in this study** |
| --- |

| **Name** | **Description** | **Sequence** |
| --- | --- | --- |
| yhaJ_BamHI_5 | *yhaJ* forward primer with 5’ in-frame *Bam*HI flank for cloning into pET28b | GGATCCATGGCCAAAGAAAGGGCATT |
| yhaJ_HindIII_3 | *yhaJ* reverse primer with 5’ in-frame *Hind*III flank for cloning into pET28b | AAGCTTTTATTTTCCGTTAAAAAGTT |
| espD_5_QPCR | qRT-PCR forward *espD* | GCGCACAAGCTATCCCTATC |
| espD_3_QPCR | qRT-PCR reverse *espD* | ACTTTCTGCGCGGAAGTATC |
| tir_5_QPCR | qRT-PCR forward *tir* | GGGATCTCGTGCGCTATTTA |
| tir_3_QPCR | qRT-PCR reverse *tir* | AGTCCAGGAACATCACTGGC |
| ler_5_QPCR | qRT-PCR forward *ler* | GACTGCGAGAGCAGGAAGTT |
| ler_3_QPCR | qRT-PCR reverse *ler* | ATCCCAGCTCTTGTAAGGTT |
| nleA_5_QPCR | qRT-PCR forward *nleA* | TAGGATGCCAAGCTGGATTT |
| nleA_3_QPCR | qRT-PCR reverse *nleA* | AGCTGTTGTTTCACCGCATT |
| nleG_5_QPCR | qRT-PCR forward *nleG* | ATAGTGAAACGGATGGTCGC |
| nleG_3_QPCR | qRT-PCR reverse *nleG* | TTCAGGACCACCATTAAGCC |
| gapA_5_QPCR | qRT-PCR forward *gapA* | GCTAACCTGAAATGGGACGA |
| gapA_3_QPCR | qRT-PCR reverse *gapA* | CACCAGCGGTGATGTGTTTA |
| ler_P1_5 | *ler* P1 promoter region forward (EMSA) | ATGTAAGCTCCTGGGGATTCAC |
| ler_P1_3 | *ler* P1 promoter region reverse (EMSA) | CAAACAACCACCTTAAAATG |
| ler_p2_5 | *ler* P2 promoter region forward (EMSA) | CATTTTAAGGTGGTTGTTTGA |
| ler_P2_3 | *ler* P2 promoter region reverse (EMSA) | CCTGCTGTAGAACTGCAATTTG |
| yhaO_promoter_5 | *yhaO* promoter region forward (EMSA) | GCGGACTGGCCATTAAAGT |
| yhaO_promoter_3 | *yhaO* promoter region reverse (EMSA) | TCGCTCAATATTTTGTAGGGCT |
| nleA_promoter_5 | *nleA* promoter region forward (EMSA) | ACAGAGCATTAGCGCAAGGT |
| nleA_promoter_3 | *nleA* promoter region reverse (EMSA) | TCACATATCCGATGTGGACAG |
| nleB_promoter_5 | *nleB* promoter region forward (EMSA) | CATTTTCCTGCAAAAGCTCA |
| nleB_promoter_3 | *nleB* promoter region reverse (EMSA) | ATGTTAATATCGCCCCGTCA |
| nleG1_promoter_5 | *nleG1* promoter region forward (EMSA) | AATCTTTGGGGCAATGGAAC |
| nleG1_promoter_3 | *nleG1* promoter region reverse (EMSA) | TCATTCCAAACACAGGGAGTC |
| nleG6_promoter_5 | *nleG6* promoter region forward (EMSA) | ACTCAGGTGAAGCACAATCG |
| nleG6_promoter_3 | *nleG6-2* promoter region reverse (EMSA) | TCAGTTTGCAGGATGTTTCA |
| Kan_control_5 | *Kan* gene (pUC57) negative control | GGCAAAAGTTTATGCATTTC |
| Kan_control_3 | *Kan* gene (pUC57) negative control | GCATTTTATCCGTACTCCTG |
